# Mapping molecules in scanning far-field fluorescence nanoscopy

**DOI:** 10.1038/ncomms8977

**Published:** 2015-08-13

**Authors:** Haisen Ta, Jan Keller, Markus Haltmeier, Sinem K. Saka, Jürgen Schmied, Felipe Opazo, Philip Tinnefeld, Axel Munk, Stefan W. Hell

**Affiliations:** 1 Department of NanoBiophotonics, Max Planck Institute for Biophysical Chemistry, Göttingen 37077, Germany; 2 Statistical Inverse Problems in Biophysics Group, Max Planck Institute for Biophysical Chemistry, Göttingen 37077, Germany; 3 Department of Mathematics, University of Innsbruck, Innsbruck 6020, Austria; 4 Department of Neuro- and Sensory Physiology, University Medical Center Göttingen, Göttingen 37073, Germany; 5 NanoBioSciences Group, Institute of Physical and Theoretical Chemistry, Braunschweig University of Technology, Braunschweig 38106, Germany; 6 Institute for Mathematical Stochastics and Felix Bernstein Institute for Mathematical Statistics in the Biosciences, University of Göttingen, Göttingen 37077, Germany

## Abstract

In fluorescence microscopy, the distribution of the emitting molecule number in space is usually obtained by dividing the measured fluorescence by that of a single emitter. However, the brightness of individual emitters may vary strongly in the sample or be inaccessible. Moreover, with increasing (super-) resolution, fewer molecules are found per pixel, making this approach unreliable. Here we map the distribution of molecules by exploiting the fact that a single molecule emits only a single photon at a time. Thus, by analysing the simultaneous arrival of multiple photons during confocal imaging, we can establish the number and local brightness of typically up to 20 molecules per confocal (diffraction sized) recording volume. Subsequent recording by stimulated emission depletion microscopy provides the distribution of the number of molecules with subdiffraction resolution. The method is applied to mapping the three-dimensional nanoscale organization of internalized transferrin receptors on human HEK293 cells.

## Abstract (web summary)

Mapping the distribution of fluorescence molecules, rather than just their emission intensity, can improve super-resolution fluorescence microscopy. Here, the authors present a general solution for rendering the number of fluorescent molecules recorded by confocal or STED microscopy.

## Graphical abstract

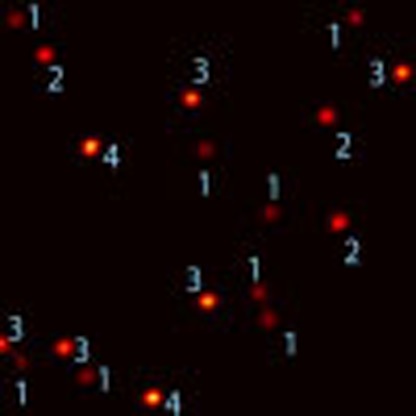

Ideally, a microscope discerns and maps all features and molecules of interest in the sample. Throughout the twentieth century it was widely accepted that any attempt to develop such a microscope would require an electron or scanning probe type of approach, because discerning features that are closer together than about half the wavelength of light would not be possible using optical lenses^1^. Today, the diffraction barrier is overcome and developing far-field fluorescence microscopy towards this aim has become realistic. The reason why modern ‘super-resolution' fluorescence microscopes or ‘nanoscopes' are able to discriminate densely packed molecules is that the discrimination of the molecules is no longer accomplished by focusing the light in use. Rather the molecules are transiently prompted into two different states, usually fluorescence ‘on' and ‘off' states, so that they are distinguishable when illuminated by the same diffraction pattern^2^.

Of major relevance to mapping the molecules is that this transient state difference can be elicited either in a spatially controlled (coordinate targeted) or in a spatially stochastic manner. The first strategy is realized in the methods called stimulated emission depletion (STED)^3^,^4^, saturated structured-illumination microscopy (SSIM)^5^ and reversible saturable optical fluorescence transitions (RESOLFT)^6^,^7^. Here a distribution of light with one or multiple intensity minima drives the molecules optically between an ‘on' and an ‘off' state, thus transferring all molecules to one of these states except those located at a defined subdiffraction-sized region around the minima. The light pattern is scanned across the sample so that every molecule eventually ends up in the narrow minimum, and hence in a state that is different from that of the nearby molecules outside this minimum.

Spatially stochastic methods such as those called photoactivated localization microscopy (PALM)/stochastic optical reconstruction microscopy (STORM) prompt adjacent molecules individually to a specific transient on-state in which the molecule is able to emit multiple fluorescence photons^8^,^9^,^10^,^11^. The multiple photons are then used for identifying the position of the molecule with subdiffraction precision. Although spatially stochastic methods can provide molecular maps^12^, counting molecules with stochastic methods is not as straightforward as it may seem. Apart from the ability to transiently assume a multiple-photon emitting on-state, other requirements need to be fulfilled to count molecules reliably. Molecules that do not emit enough photons in the on-state—or do not assume this state at all—are missed out completely. Furthermore, some molecules will occupy this on-state repeatedly, and be counted multiple times. For these reasons, fluorophores that assume the on-state only once, such as mEos2 (ref. 12), are preferred. However, these fluorophores allow only a single super-resolution recording, meaning that molecular mapping is not repeatable. Last but not least, counting molecules one by one requires extended recording times during which the molecules must not move.

In contrast, STED microscopy works with standard fluorophores that do not require photoswitching between metastable states, because the on–off-based super-resolving ability is accomplished with the fluorophore's basic ground and fluorescent state. Being not based on single-molecule detection and by registering all molecules from a given coordinate simultaneously, STED microscopy provides a potential speed advantage, but the very same fact makes molecule counting more challenging. In principle, one can extract the number of registered molecules from the magnitude of the fluorescence signal, but in practice this approach is compromised by variations in molecular brightness due to the local environmental heterogeneity. In addition, in most cases, the local brightness of individual molecules remains elusive, because the sample does not contain isolated molecules, or such molecules are simply not found. A reliable method to reveal the number of molecules in coordinate-targeted nanoscopy is very desirable but still missing. Fortunately, the number of molecules is also encoded in the photon emission statistics, specifically in the number of photons that are emitted simultaneously. Measuring the simultaneous arrival of fluorescence photons has been shown to identify individual fluorophores^13^,^14^, improve the resolution of fluorescence microscopy^15^,^16^ and analyse picked single clusters in space^17^,^18^,^19^.

Here we explore the same effect of simultaneous photon arrivals in imaging mode and establish a theoretical model followed by a standard reconstruction procedure to retrieve the number of molecules with diffraction unlimited resolution.

## Results

### Molecular mapping model

Mapping the molecule distribution can be reduced to finding a function *n*(*r*) describing the number of molecules at coordinate *r*±Δ*r*/2, with Δ*r* denoting the spatial interval in which molecules can assume the on-state. Δ*r* is equivalent to the spatial resolution given by the full-width at half-maximum of the effective point spread function (PSF) *h*(*r*) of the imaging process. Excitation is accomplished with illumination pulses much shorter than the fluorescence lifetime so that each molecule is excited at most once per pulse (see Supplementary Note 1). Molecules located at the same coordinate *r* are assumed to have the same brightness *p*(*r*), which is the probability of a molecule at the focal centre to yield a detected photon. *p*(*r*) takes into account the excitation intensity, the absorption cross-section, the fluorescence quantum yield and the collection efficiency of the setup. The ideal case *p*(*r*)=1 is reached if the molecule contributes with a detected photon for every pulse. If *p*(*r*) is temporally constant, the probabilities *f*_*i*_(*r*), *i*=1, 2, of recording *i* photon detection events per illumination pulse at coordinate *r* are:


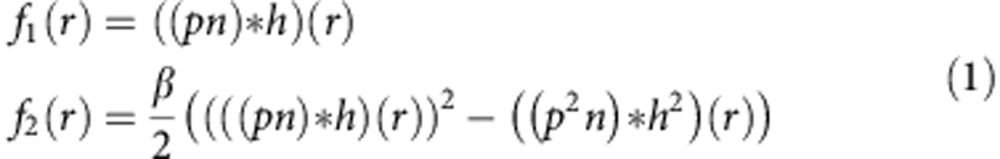


*β* considers the detection geometry (see Supplementary Note 1) and ‘*' denotes the convolution operation. *f*_2_ is given by the probability of detecting two photons from any molecule minus the ‘antibunching term' (*p*^2^*n*)*h^2^, which accounts for the probability that the two photons stem from the same molecule. *f*_2_ depends quadratically on *p*(*r*), making it a critical parameter. If (np*h)(*r*)<<1 does not hold, for example, when the number of simultaneously recorded molecules is too large, equation (1) must be extended by terms describing coincidence events of order >2.

The strategy is now to estimate *f*_1_ and *f*_2_ by detecting the number of one- and two-photon events divided by the number of pulses applied. The desired *n*(*r*) is ultimately extracted by fitting the measured *f*_1_ and *f*_2_ with the model from equation (1). This is accomplished by a fast proximal gradient algorithm^20^ that minimizes the squared distance between the model and the experiment, while penalizing strong variations of *p*(*r*) (Supplementary Note 1).

**Figure 1:**
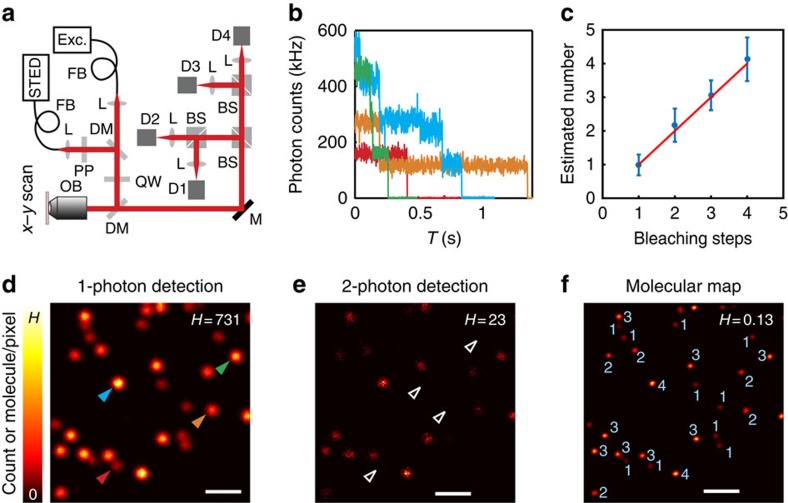
Mapping molecule distributions in far-field fluorescence microscopy through analysis of coincident photon detection. (**a**) Experimental confocal/STED setup equipped with four independent detection channels. BS, 1:1 beam splitter; DM, dichroic mirror; D_*i*_, *i*^th^ detector, *i*=1, 2, 3 and 4; FB, fibre; L, lens; M, mirror; OB, objective lens; PP, phase plate; QW, quarter wave plate. (**b**-**f**) Double-stranded DNAs (dsDNA) labelled with up to four ATTO 647N was sparsely immobilized on a glass surface. The numbers of dye molecules in each dsDNA were established by analysing the distribution of coincident photon detection, which was recorded by confocal microscopy. (**b**) Fluorescence bleaching steps of single dsDNAs. The corresponding dsDNAs are indicated in **d** by triangles with the same colours. (**c**) Comparison of number of dye molecules (mean and s.d.) derived from photon coincidence recordings with the number of bleaching steps of the same single dsDNAs. Red line: *y*=*x*. The number of molecules from photon statistics is slightly higher than that from bleaching steps due to the bleaching during scanning. The statistics of each point were based on 39–108 dsDNAs. (**d**,**e**) An example image pair of one- and two- photon detection events of immobilized dsDNA labelled with up to four ATTO 647N molecules. The positions where single dye molecules were located are indicated by open triangles in **e**. (**f**) The established map of the number of ATTO 647N molecules on each dsDNA from **d** and **e**. The numbers indicate the numbers of dye molecules in each dsDNA. H is the maximum value of the pseudocolour intensity scale, meaning counts in **d** and **e** and the number of molecules in **f** at each pixel. Original pixel size in **d** and **e** is 20 nm and is binned to 40 nm for better visualization. Scale bars, 1 μm.

The main cause of deviation between the model and the measurement is shot noise. Interestingly, the relative standard deviations (RSTDs) of the estimated number *n*_est_ and the brightness *p*_est_ of molecules in a single cluster can be derived analytically as 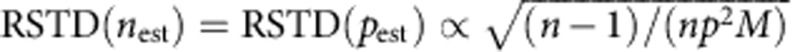 (Supplementary Note 1), which was confirmed by simulations (Supplementary Fig. 1). *M* is the number of excitation pulses. Note that for increasing *n*, RSTD(*n*_est_) does essentially not depend on *n*. The calculations and simulations suggest that the RSTD of the estimations on both *n* and *p* can be below 20% under conditions provided by routinely used synthetic fluorophores, such as ATTO 647N and Abberior STAR 635P (Supplementary Fig. 1). Therefore, we built a stage scanning confocal microscope with an optional STED beam^21^, and the detection path was split into four equivalent units (Fig. 1a) so that most of the simultaneously arriving photons could be identified as one-, two-, three- and four-photon detection events.

### Molecular mapping in confocal microscopy

To validate our prediction, we utilized double-stranded DNA (dsDNA) that is easy to synthesize and label in a controlled manner. Our first sample consisted of dsDNA labelled with up to four ATTO 647N or two Abberior STAR 635P fluorophores, sparsely immobilized on a glass surface. A pixel dwell time of 300 μs (∼3,000 excitation pulses with 10 MHz) and 20-nm-sized pixels of the confocal recording were adequate to collect 6,000–7,500 photons per fluorophore and a reduction of ∼30 per fluorophore in two-photon detection events because two-photon emission cannot come from the same molecule (Fig. 1d,e). Thus, according to our calculations and simulations, we mapped the number of fluorophores in each dsDNA with an uncertainty of ∼20% or less (Fig. 1c; Supplementary Note 1). For dsDNA containing only one molecule, no two-photon detection events were detected (Fig. 1f,e).

Distributing the dsDNA sparsely on the surface is not required for our method, but it facilitated counting the number of fluorophores on the dsDNA by bleaching them down individually. The number of individual bleaching steps matched the established number of fluorophores with a RSTD of <20% (Fig. 1b; Supplementary Fig. 2), which lies within the range of the theoretical uncertainty of our estimation. Note that our method is more versatile than counting by bleaching, because we do not require observing abrupt changes in brightness, which is unreliable when two or more molecules bleach simultaneously or when they blink. Besides, counting by photon statistics can be applied repeatedly because the molecules stay intact except for gradual bleaching during multiple scans (Supplementary Fig. 3).

### Molecular mapping in STED and confocal microscopy

On STED illumination, the apparent size of the dsDNA becomes smaller as the resolution of the system improves. At the same time, the number of photon detection events per molecule decreases and the uncertainty (RSTD) of the molecule counting increases (Supplementary Fig. 4). To keep the uncertainty below 30%, either the resolution gain has to be limited to only ∼1.5 times corresponding to ∼75 pJ STED pulse energy (Supplementary Fig. 4) or the measurement time has to be prolonged to collect sufficient photon events. However, long measurement time leads to loss of fluorophores by bleaching before enough photon detection events can be obtained. Fortunately, the bleaching rate in confocal recordings is as low as ∼3% per scan for ATTO 647N (Supplementary Fig. 3). This allows us to perform a STED recording right afterwards so that we can exploit the photon statistics of both recordings synergistically. Then, equation (1) extends to


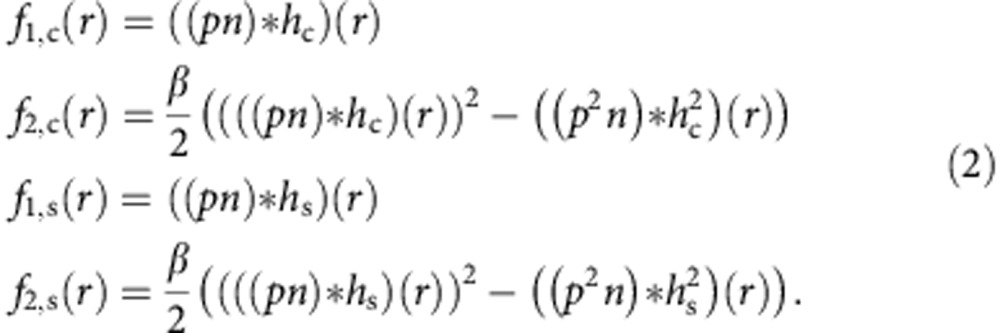


Here, *f*_*i*,c/s_(*r*) means *i* (*i*=1, 2) photon detection probability of confocal/STED microscopy, with *h*_c/s_ denoting the corresponding effective PSF. To recover *n* and *p*, the fitting is now conducted by minimizing the distance from the model in equation (2) to the *i* photon detection events (normalized to the overall illumination pulses) simultaneously in both confocal and STED measurements (Supplementary Note 1). The combined method yields the molecule numbers at the STED microscopy resolution of <50 nm (Supplementary Fig. 5).

**Figure 2:**
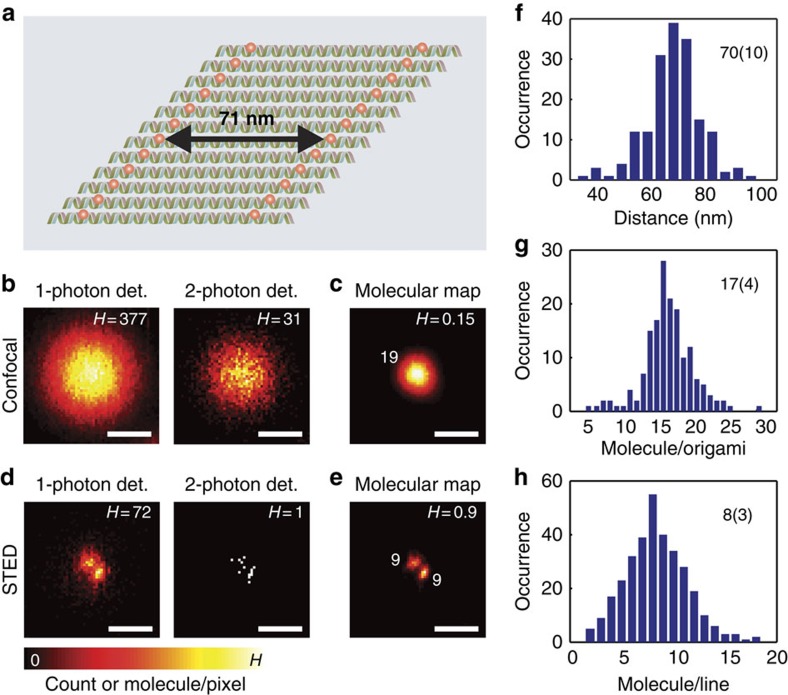
Mapping the number of molecules in a controlled biological sample. DNA origami labelled with ATTO 647N molecules were immobilized on the surface and measured with confocal and STED microscopy. (**a**) Sketch of one DNA origami. Red dots represent the locations where ATTO 647N molecules can be conjugated. Each DNA origami can accommodates up to 24 fluorophores (12 in each line). (**b**) Confocal one-photon (left) and two-photon (right) detection images. (**c**) Map of the number of ATTO 647N molecules on single DNA origami calculated based on **b**. (**d**) STED one-photon (left) and two-photon (right) detection images. (**e**) Map of the number of ATTO 647N molecules on single DNA origami based on **b** and **d**. (**f**) The histogram of the distance between the two fluorescent lines in one DNA origami from the reconstructed images. (**g**,**h**) Histograms of the numbers of ATTO 647N molecules in one DNA origami (**g**) and one line of DNA origami (**h**) from the extracted number maps. H is the maximum value of the pseudocolor intensity scale and specified on the top-right corner of the corresponding images, meaning counts in **b, d** and the number of molecules at each pixel in **c** and **e**. The numbers in the histograms (**f**,**g,h**) are the mean values and s.d. Scale bars, 200 nm.

Encouraged by these results, we turned to DNA origami, which is an excellent scaffold to attach a defined number of molecules at defined positions within a diffraction-limited volume^22^. We designed a DNA origami sheet containing up to 24 fluorophores, arrayed in two lines of up to 12; the distance between the lines was adjusted by design to 71 nm (Fig. 2a). Due to the limited folding efficiency of DNA, the number of fluorophores per DNA origami was expected to be lower on average, namely ∼19 (ref. 22). While confocal microscopy could not resolve the lines within the DNA origami (Fig. 2b), it allowed retrieving the number of molecules by recording two-photon detection events (Fig. 2c; Supplementary Fig. 6). Thus, we identified 13–21 dye molecules per cluster (Fig. 2g), which is in good agreement with the expected range of numbers^22^ (Supplementary Fig. 7). The fivefold improved lateral resolution provided by STED resolved the lines reliably (Fig. 2d; Supplementary Fig. 6). Analysing the confocal and STED image pairs resolved the molecular distribution in the DNA origami sheet (Fig. 2e; Supplementary Fig. 6). We found a spacing of 70±10 nm (mean±s.d.) between the lines, which is in excellent agreement with the expected value of 71 nm (Fig. 2f), and 5–11 molecules on each line (Fig. 2h).

### Molecular mapping in cells in 3D

As indicated in equations (1) and (2), our method is not restricted to two-dimensional specimen. The extension to three dimension (3D) is rather straightforward (Supplementary Fig. 8). We chose a step size of 100 nm between the stacks, which is below the axial full-width at half-maximum of the PSF (∼570 nm). Since each molecule was illuminated in several stacks, the pixel dwell time was shortened to 100 μs (∼1,000 illumination pulses) to reach the same estimation uncertainty (∼20% RSTD) as in the two-dimensional cases. This 3D extension facilitated applications to (the interior of) cells, here specifically to counting of transferrin receptor (TfR) molecules in human embryonic kidney 293 (HEK293) cells. The TfR molecules were labelled by an ATTO 647N-conjugated DNA aptamer, that is, by labelled oligonucleotides. We chose aptamer labelling^23^,^24^ because one can attach a single fluorophore per aptamer in a controlled way. Furthermore, the aptamers are designed to bind to their target in a one-to-one stoichiometry, making quantification straightforward.

**Figure 3:**
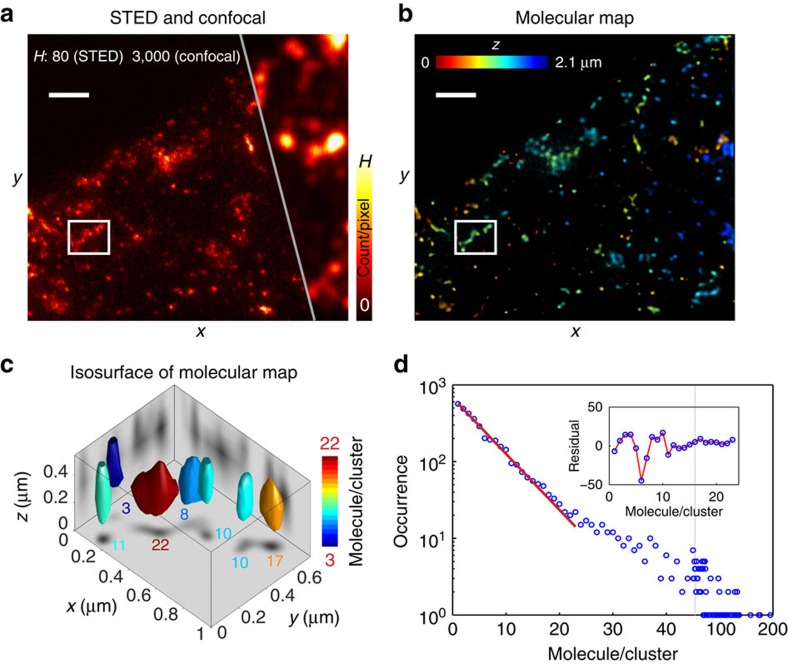
Mapping the number of transferrin receptors (TfR) in HEK293 cells. Living cells were incubated with ATTO 647N-conjugated anti-TfR aptamer c2 for 60 min. After incubation, excess aptamer molecules were washed off and cells were chemically fixed. Stained receptors were imaged by confocal and STED microscopy (see Methods for details). (**a**) Confocal and STED images (raw data): summation projection along the axial dimension (0.9 μm). H is the maximum value of the pseudocolour intensity, meaning counts. (**b**) 3D molecular map generated by photon statistics of both confocal and STED recordings. Colours represent the axial (z) position. (**c**) Isosurfaces of the molecular map (corresponding to the box region in **a** and **b**). The isosurfaces embrace 70% of the overall molecules in the corresponding regions. The grey surfaces are the summation projections of the number of molecules to the corresponding dimensions. Colours represent the number of molecules in the corresponding region. The numbers of molecules in the corresponding segmentations are indicated on the z-projection plane. (**d**) The histogram of the number of molecules in the recognized separated clusters in the generated molecule map. Identification of the clustering is performed with built-in watershed algorithm provided in MATLAB. The red line is the exponential distribution fit to the occurrences of up to 24 molecules in each spot (blue circle). Clusters of TfRs with more than 24 molecules are not considered due to potential overlapping of multiple clusters under the given resolution of the STED microscope. Inset: the residual of the fit from the experimental observation. Scale bars, 1 μm.

In confocal recordings, the TfRs are largely overlapping in space and their distribution is not resolvable (Fig. 3a; Supplementary Fig. 9; Supplementary Movie 1). However, the additional two-photon detection information reveals that the average density of TfRs inside the HEK293 cells is ∼62 nM. Considering the average volume of HEK293 cells, ∼2.7 pl (Supplementary Fig. 10), the number of internalized TfRs in each cell is ∼100 k, which is also confirmed by quantitative western blots (∼110 k, Supplementary Fig. 10). In addition, our method determines the molecular density variation of internalized TfRs among the observation regions as ∼18 nM or ∼29%, which is not possible by conventional ensemble measurements, such as quantitative western blot. With the improved resolution in STED microscopy, most intracellular TfRs can be visualized as separated clusters with a density of 4.4±2.6 fl^−1^ (Fig. 3a; Supplementary Movie 1). Again, the recorded coincidence photon detection of both confocal and STED measurements generates the molecule distribution at nanoscale within the cells (Fig. 3b,c; Supplementary Movie 1). Interestingly, the number of TfRs in the isolated clusters follows an exponential distribution with an expectation of ∼ 6.0±1.9 per cluster (Fig. 3d; Supplementary Fig. 11). It also indicates that one cluster has the capacity to accommodate >20 TfRs since we do not observe clear deviation from the exponential distribution up to 20 (Fig. 3d).

## Discussion

The quadratic dependence of *f*_2_ on *p*(*r*) calls for relatively high excitation intensities, but since high intensities increase blinking and bleaching, we settled for ∼0.1 pJ per excitation pulse giving ∼60% of the maximal emission (Supplementary Fig. 12). The pulse repetition rate was kept at 10 or 20 MHz to avoid the fluorophores accumulating in metastable dark (blinking) states^25^. Under the so-called ROXS buffer conditions^26^, ∼7.6% of the illuminated fluorophores were trapped in a ∼ 9-μs long dark state (Supplementary Fig. 13). Transient dark states reduce the molecular brightness but do not really affect the counting. The average distance between the fluorophores in the DNA origami and dsDNA was 6 nm, which probably is the main reason why we did not observe fluorescence fluctuations induced by intermolecular interactions.

Since the RSTD remains nearly independent of *n* for *n*>5 (Supplementary Fig. 1), the maximum number of molecules in the excitation volume has not reached the theoretical limit in our experiments. In practice, the detection electronics suffers from saturation before the counting limit is reached, which can be alleviated by reducing the illumination repetition rate while increasing the measurement time or increasing the number of detectors and considering higher orders of coincidence. Also, owing to the fact that our method considers variations of molecular brightness at effective PSF scale and larger, we can investigate the heterogeneity in both the number of molecules but also the molecular brightness. For example, the molecular brightness of multiple Cy5 or ATTO 647N labelled dsDNA in the same sample can be discerned as 0.013±0.004 (mean±s.d.) and 0.022±0.04, respectively, which is mainly due to the differences in their properties, for example, quantum yield and cross-section (Supplementary Fig. 14).

Our study reveals that counting the molecules in a confocal recording and allocating them using STED is a favourable strategy, because the counting accuracy benefits from increased resolution (small Δ*r*) only if the number of simultaneously recorded molecules becomes lower, for example, <5. Our study also suggests that collecting the emitted fluorescence from both sides of the sample will facilitate the molecule counting in a pure STED mode at better resolution. Note that the effective PSF describing the imaging by simultaneous *m*-photon detection^15^ or *m*^th^-order antibunching^16^ are narrower by 
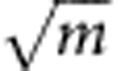
 than that describing regular one-photon detection, meaning they provide improved resolution (Supplementary Fig. 15). In any case, both confocal and STED imaging benefit from coincident photon detection (Supplementary Fig. 15).

Since it does not require metastable on- or off-states and the molecules can be counted in groups at once, our counting is faster than that based on single molecules, especially on a small region of interest. In our DNA origami experiment, counting in a 10 × 10 μm^2^ area lasted <60 s or 1 × 1 μm^2^ within 1 s. The recording time of a big field of view can be cut down using many focal spots in parallel^27^.

STED microscopy is viable with standard fluorophores that do not require photoswitching. However, the need to analyse photon coincidences calls for many emissions of the dyes in use and hence for relatively stable dyes. Since it does not strictly rely on a particular on–off transition, our approach should also be applicable to other types of coordinate-targeted super-resolution microscopy besides STED. In any case, a far-field optical method that rapidly and repeatedly maps the distribution of molecules with nanometric resolution should become a standard tool for exploring cellular physiology at its most fundamental level.

## Methods

### Microscope setup

Our molecule mapping microscope is equipped with a 635-nm-pulsed diode laser (LDH-D-C-635, PicoQuant, Berlin, Germany) with 100-ps pulse width for fluorescence excitation, and a mode-locked titanium–sapphire laser operating at 770 nm for STED (Mira900, Coherent). The repetition rate of the STED laser is 76 MHz and the initial 150-fs pulse width is stretched to about 200 ps using an optical fibre. The excitation and STED laser pulses are synchronized, but the excitation is triggered only at every 4th or 7th pulse to reduce the repetition rate to ∼20 or 10 MHz, respectively. The focal STED doughnut was created by passing the STED laser beam through a polymer vortex phase plate (RPC Photonics, Rochester, New York, USA) and feeding it into a 1.4 numerical aperture (NA) objective lens (HCX PL APO 100X 1.40–0.70, Leica Microsystems, Mannheim, Germany). The fluorescence was collected by the same objective lens and filtered out by passive dichroic mirrors and filters (zt 625–745 rpc, F72-750P and F47–686, AHF Analysentechnik AG, Tuebingen, Germany). The fluorescence beam was split into four channels by three 50:50 beam splitters, coupled into four multimode fibres and detected by four avalanche photodiodes (SPCMAQR-13-FC, PerkinElmer Optoelectronics, Waltham, Massachusetts, USA). The fibres acted as confocal pinholes and were aligned to the focal excitation maximum.

### Image acquisition and analysis

The scanning, data acquisition and analysis software (Supplementary software 1) were coded in C++ and MATLAB (The Mathworks Inc., Natick, Massachusetts, USA). A three-axis piezo table (NanoMax, Thorlabs Inc., Newton, New Jersey, USA) was used to raster scan the samples, controlled by a multifunction analogue output computer card NI PCI-6731 (National Instruments, Austin, Texas, USA). Data acquisition were performed with a time-correlated single-photon counting board in absolute timing mode (DPC-230, Becker & Hickl GmbH, Berlin, Germany). We considered detection events in a 12-ns time window starting at the beginning of the excitation pulse.

### Double-stranded DNA for calibration

The dsDNAs with multiple ATTO 647N, Cy5 or Abberior STAR 635P molecules are formed by annealing from single-stranded DNAs (ssDNA), which were purchased directly from IBA (Göttingen, Germany), by heating the mixture of complementary ssDNAs to 95 °C and cooling it down to room temperature gradually in 45 min. The sequences of ssDNAs with four ATTO 647N molecules: biotin 5′-CGCCTATCCATAAACGAGGAGGACGCCTATCCATAAACGAGGAGGACGCCT ATCCATAAACGAGGAGGACGCCTATCCATAAACGAGGAGGA-3′ and 5′-TCCTCC TCGTT(ATTO 647N)ATGGATAGGCG-3′; the sequences of the ssDNAs with five Cy5 molecules: biotin 5′-ACACGACTTCTCCTCTCTCTCACGTTCCTTACCTACACCATCTTTACC TACTTACCCCACATCACTATCCCTTCTCATTCCTTTA-3′ and Cy5 5′-TAAAGG AATGAGAAGGGATAGT(Cy5)GATGTGGGGTAAGTAGGTAAA(Cy5)GATGGTGTAGG TAAGGAACGT(Cy5)GAGAGAGAGGAGAAGTCGTGT-3′; and the sequences of the ssDNAs with two Abberior STAR 635P: (biotin) 5′-TTATTCCTGTAGTATATGGCAATGAAA TTAT-3′ and 5′-ATAATTTCATTGCCATATACTACAGGAATAA-3′. The immobilization is performed by streptavidin–biotin linkage to the biotinylated bovine serum albumin (BSA; Sigma-Aldrich, St Louis, Missouri, USA)-coated glass surface. In brief, the coverslip was cleaned with 2% Hellmanex II (Hellma GmbH, Müllheim, Germany) and washed with PBS, pH 7.4, and, then, incubated with 1 mg ml^−1^ biotinylated BSA for 30 min. After another washing step with PBS buffer, a solution of 0.5 mg ml^−1^ streptavidin (Roche Diagnostics, Mannheim, Germany) was used to incubate the coverslip for 30 min. Finally, the coverslip was washed again with PBS and was used for immobilization of the dsDNA (100 pM). ROXS buffer was used to increase the photostability^26^.

### Immobilized DNA origami

The DNA origami sheet was assembled from a long single-stranded scaffold strand from M13mp18 phage and ∼200 staple strands^22^. The DNA origami is rectangular in shape and designed with two lines of ATTO 647N dyes and five biotin molecules for immobilization. Each line of the dye molecules has up to 12 ATTO 647N molecules and the distance between them is 71 or 41 nm. The immobilization of the DNA origami is the same as the dsDNA via biotin–streptavidin interaction. ROXS buffer was used for measurements^26^.

### Cell culture

HEK293 cells were cultured in high glucose (4.5 g l^−1^) DMEM supplemented with 10% fetal calf serum, 4 mM L-glutamine and 100 U ml^−1^ of each penicillin and streptomycin. For imaging, cells were grown on poly-L-lysine-coated glass coverslips and typically stained 16 h after seeding.

### Cellular volume

HEK293 cells seeded on glass coverslips were chemically fixed in PBS supplemented with 4% paraformaldehyde (PFA) for 30 min at room temperature. Quenching of the remaining reactive formaldehyde groups was performed by incubation for 15 min in PBS supplemented with 0.1 M glycine and 0.1 M NH_4_Cl. After a brief wash with PBS, cell membranes were stained for 10 min at room temperature with 1 μg ml^−1^ octadecyl rhodamine B (Molecular Probes, Eugene, OR, USA) in PBS followed by 10-min incubation with 250 μM nuclear staining dye SYTOX green (Invitrogen, Carlsbad, California, USA). Finally, cells were rinsed in PBS and mounted in Mowiöl mounting media (6 g glycerol, 6 ml deionized water, 12 ml 0.2 M Tris buffer pH 8.5, 2.4 g Mowiöl 4–88, Merck KGaA, Darmstadt, Germany). Confocal images were obtained using a TCS STED SP5 fluorescence microscope (Leica Microsystems GmbH), equipped with a 1.4 NA/63 × STED objective (Leica). Confocal sections with pinhole diameter set to 1 Airy disc were acquired to cover the complete volume of individual cells. Total cellular volumes and nuclear volumes were calculated by manual selection of cellular or nuclei contour (in focus) for every optical section (using ImageJ). The calculated areas of each slide were multiplied by the section thickness (0.797 μm) and summed up to obtain the volumes of the whole cell and nucleus. Cytosolic volume was obtained by subtracting the nuclear volume from the total cell volume. Average cell volume was obtained from 10 individual cells.

### Immunostainings

HEK293 cells on poly-L-lysine-coated coverslips were fixed in 4% PFA for 45 min at room temperature, washed with PBS. Remaining formaldehyde was quenched with 0.1 M glycine and NH_4_Cl in PBS for 15 min. To perform a complete immunostaining that also labels the internal epitopes, coverslips were incubated with 2% BSA and 0.1% Triton X-100 in PBS for 15 min at room temperature. To detect only the TfRs present at the cell surface, cells were incubated in 2% BSA in PBS (without detergent). Primary antibody anti-TfR (ab1086, Abcam, Cambridge, UK) was used at a final concentration of 10 μg ml^−1^ in PBS with 2% BSA for 60 min at room temperature. This monoclonal mouse antibody was chosen since it targets an extracellular epitope of the TfR, allowing us to perform the staining of surface molecules. After thorough washing steps, primary antibody was detected by Cy3-coupled anti-mouse secondary (Dianova, Hamburg, Germany). Finally, coverslips were rinsed in PBS and mounted in Mowiöl (see above). Epifluorescence images were acquired with an Olympus IX71 microscope equipped with 0.75 NA/40 × objective and an Olympus F-View II CCD camera (all from Olympus, Hamburg, Germany). Image intensities of individual cells were measured by manual selection of cellular contour (ImageJ). Average intensities for surface and complete immunostaining were obtained from three independent experiments with 30–60 cells analysed per experiment.

### Aptamer staining

Aptamer stainings were performed as described earlier^23^. Briefly, c2 aptamer anti-TfR^24^ was conjugated to ATTO 647N fluorophore. The efficiency ratio of dye to aptamer coupling was ∼1, as determined by spectrophotometry at 260 and 644 nm. Directly labelled aptamers were mixed with PBS containing 5 mM MgCl_2_, heated up in a thermocycler to 75 °C for 3 min, and then cooled to 20 °C at a rate of 1 °C min^−1^ to achieve the active conformation/folding of the aptamer. HEK293 cells were washed briefly with pre-warmed Ringer buffer (124 mM NaCl, 5 mM KCl, 2 mM CaCl2, 1 mM MgCl2, 30 mM D-glucose and 25 mM HEPES, pH 7.4) and incubated in cell medium (see above) for 30 min at 37 °C with 1 μM of ∼5 kDa dextran sulfate and 1 mg ml^−1^ of sheared salmon sperm DNA (Invitrogen) as pre-blocking agents. Cells were incubated in 60 μl of aptamer c2 at a concentration of 0.1 pmol μl^−1^ (∼3.6 × 10^12^ molecules) for 60 min (saturating staining) at 37 °C in complete medium supplemented with 1 mg ml^−1^ of sheared salmon sperm DNA. Cells were finally washed extensively with ice-cold PBS and fixed with 4% PFA as above. Following quenching of the fixative, cells were briefly rinsed and left in ROXS buffer^26^ for imaging.

### Quantitative western blotting

HEK293 cells were collected and lysed at a concentration of 2 × 10^7^ cells per ml in RIPA Buffer (Sigma-Aldrich) that was complemented with 1 mM EDTA and protease inhibitors (1 μg ml^−1^ aprotinin, 1 μg ml^−1^ leupeptin, 1 μg ml^−1^ pepstatin A and 100 μg ml^−1^ PMSF) using a cell scraper. For further homogenization, the lysates were vortexed, sonicated and passed through a 1-ml syringe fitted with a 23-gauge needle. After quantification of the protein concentration, cell lysates were snap-frozen in liquid nitrogen and stored at −80 °C. For protein separation, cell lysate samples were mixed with SDS sample buffer (50 mM Tris, 4% SDS, 0.01% Serva Blue G, 12% glycerol and 2% β-mercaptoethanol) and subject to gel electrophoresis using 10% denaturing Tris/Tricin SDS polyacrylamide gels in a discontinuous buffer system (anode buffer containing 200 mM TRIS, pH 8.9 and cathode buffer containing 1% SDS, 100 mM Tricin, 100 mM TRIS, pH 8.25). Following the gel electrophoresis, western blotting onto nitrocellulose membrane and immunolabeling were performed as described before^28^. Primary antibody incubation was performed overnight at 4 °C with rabbit anti-TfR primary antibody (HPA028598, Sigma-Aldrich). For detection of the primary antibody, anti-rabbit secondary antibody (IRDye 800 CW, LI-COR Biosciences, Lincoln, Nebraska USA) was used. Blots were imaged using the LI-COR Odyssey Imaging System at highest sensitivity. The background-corrected intensities for each band were measured using a custom-written routine in Matlab. For quantification, *in vitro* expressed recombinant full-length human TfR (Novus Biologicals, Littleton, Colorado, USA) was used as a standard. To create a standard curve, the recombinant protein was prepared in concentrations increasing from 0 to 50 ng. Fetal calf serum was added to the recombinant protein in amounts matching the total protein concentration in the HEK293 cell lysate to have equal total protein concentration in all lanes. Linear regression was applied to the linear portion of the standard curve to determine the absolute amount of the TfRs in the cell lysate. Apparent molecular weight of the protein (98 kDa) including the post-translational modifications was preferred in the calculations rather than the theoretical molecular weight of the peptide sequence.

## Additional information

**How to cite this article**: Ta, H. *et al.* Mapping molecules in scanning far-field fluorescence nanoscopy. *Nat. Commun.* 6:7977 doi: 10.1038/ncomms8977 (2015).

## Supplementary Material

Supplementary Information Supplementary Figures 1-15, Supplementary Note 1 and Supplementary References

Supplementary Movie 1 A 3D molecular map of the TfR (stained with aptamer in HEK293 cells) is reconstructed from the photon statistics of confocal and STED measurements. Isosurfaces of the molecular map which embrace 70% of the overall number of molecules in the corresponding cluster are plotted in the animation. The cluster segmentation is performed with the built in watershed function in MATLAB. 0 - 5 s: reconstruction from the photon statistics of confocal recording (Red). 6 - 11 s: reconstruction from the photon statistics of confocal and STED recording (Cyan). 19 - 31 s: reconstruction from the photon statistics of confocal and STED recording with color code representing the number of molecules in each cluster. The full region is 8 x 8 x 2 μm.

Supplementary Software 1 This software package contains the algorithm for molecular counting based on photon statistics in Confocal and STED microscopy.
