# Supplementary material for: Mapping molecules in scanning far-field fluorescence nanoscopy
Source: Nat Commun. 2015 Aug 13;6:7977. doi: 10.1038/ncomms8977 (PMC4557268; doi:10.1038/ncomms8977)
Supplement: Supplementary Information — Supplementary Figures 1-15, Supplementary Note 1 and Supplementary References [file ncomms8977-s1.pdf]

# Supplementary Figures

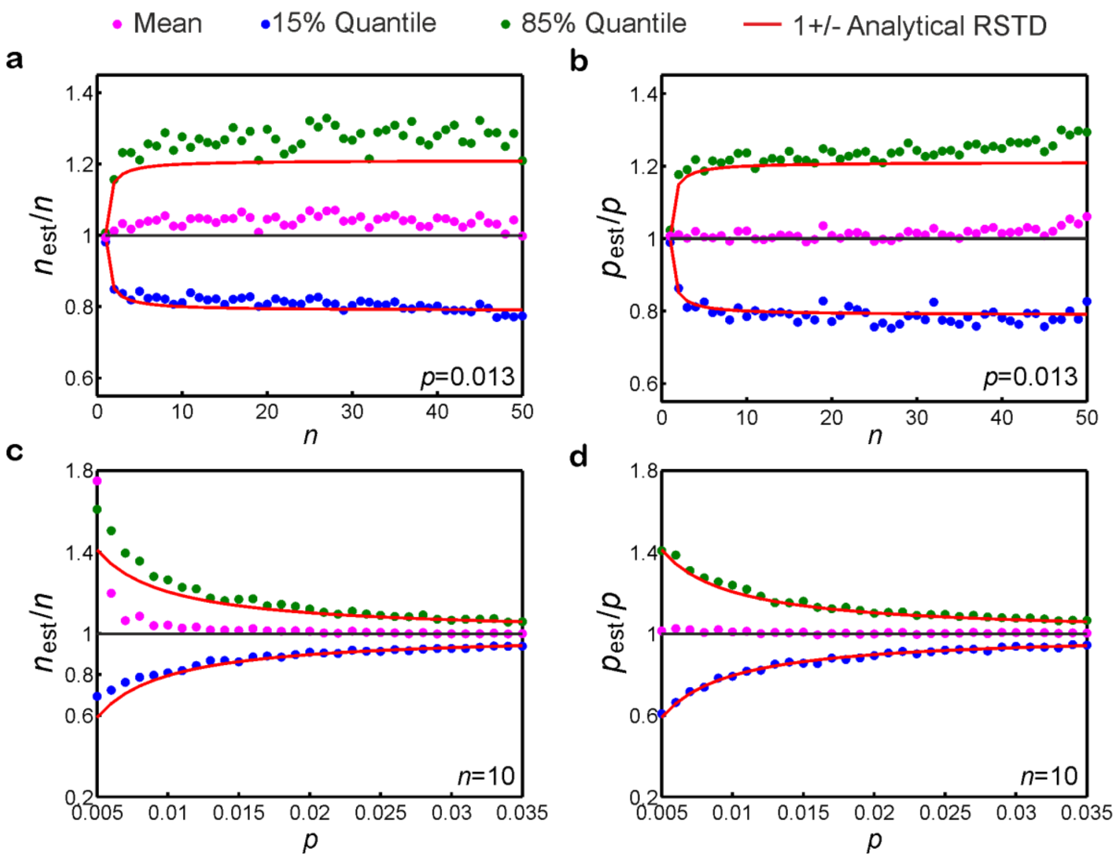


**Supplementary Figure 1: Estimation of the error of the number and brightness of molecules in a single cluster; Simulation**

(**a**,**c**) Relative estimated numbers of molecules $n_{\mathrm{est}}/n$; (**b**,**d**) relative estimated molecular brightness $p_{\mathrm{est}}/p$. $n$ and $p$ are the simulated numbers of molecules and molecular brightness. Molecules in a single cluster share the same brightness. (**a**,**b**) the number of simulated molecule $n$ varies (from 1 to 50) while keeping the molecular brightness constant $p = 0.013$. (**c**,**d**) the simulated molecular brightness $p$ varies (from 0.005 to 0.035) while keeping the number of molecules constant $n = 10$. Dots: the mean estimated values (magenta), the 15% (blue) and 85% (green) quantiles of the distribution of the values; Lines: $1 \pm$ analytically derived relative standard deviation (RSTD) of the estimated value (red) (Supplementary Note). For each condition, the simulation was repeated 500 times. The number of illumination pulses is 25,000 (**a**,**b**) and 40,000 (**c**,**d**). The full width at half maximum (FWHM) of the point spread function (PSF) is 240 nm FWHM with 48 nm pixel size.

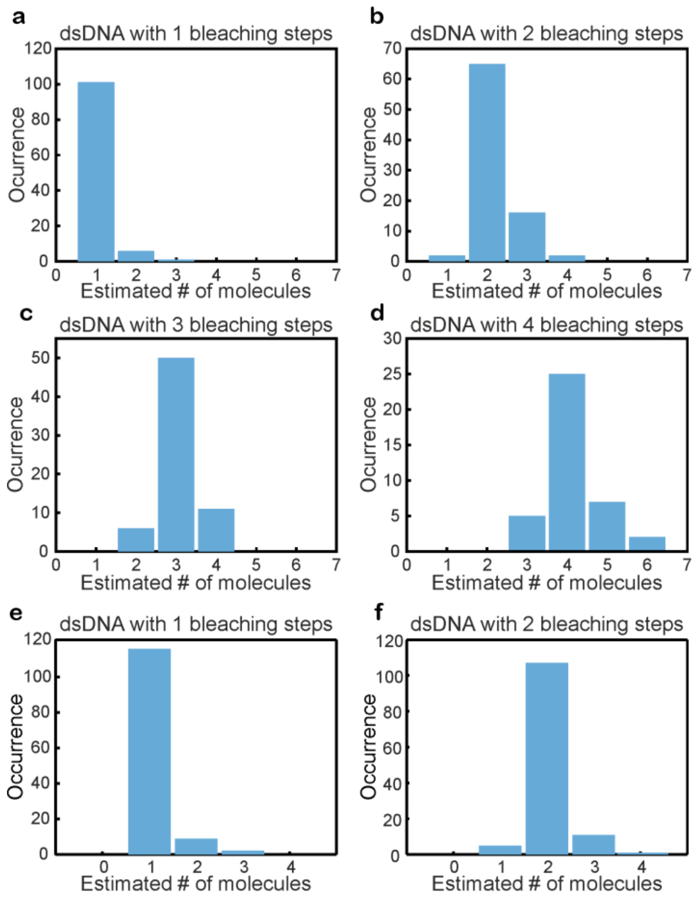


**Supplementary Figure 2: Comparison of the estimated number of molecules obtained with photon statistics or by analyzing bleaching steps**

Double stranded DNA (dsDNA) conjugated with up to 4 ATTO 647N or 2 Abberior STAR 635P was sparsely immobilized on a coated cover slip (see Methods). The estimation based on the detection of simultaneous photons was performed on the confocal scanned images with 50 nm pixel size and 1 ms pixel dwell time. Each dsDNA was localized by the confocal image and then moved into the focus individually for time-resolved fluorescence intensity measurement until all fluorophores were bleached. Histograms of the estimated number of molecules of each dsDNA are plotted: **a**, **b**, **c** and **d** correspond to dsDNA labelled with ATTO 647N with 1, 2, 3 or 4 bleaching steps respectively. **e** and **f** correspond to dsDNA labelled with Abberior STAR 635P with 1 or 2 bleaching steps respectively.

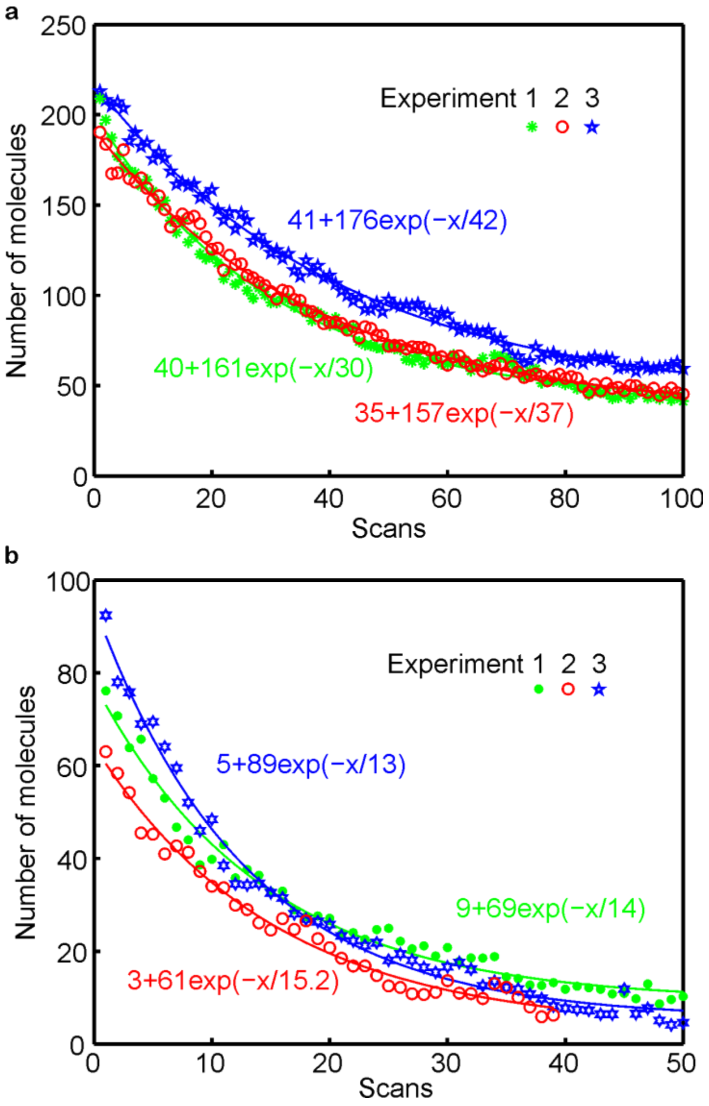


**Supplementary Figure 3: Bleaching rate under multiple confocal scans**

Immobilized dsDNA labelled with ATTO 647N (**a**) or Abberior STAR 635P (**b**) is scanned with confocal microscopy. The overall number of molecules is determined by photon statistics for each scan. The overall number of molecules in the scanning area decrease gradually from scan to scan due to bleaching. The solid lines are the exponential fitting curves to the estimated number of molecules from 3 experimental measurements with the fitting model and the resulting parameters indicated by the text in the same colors.

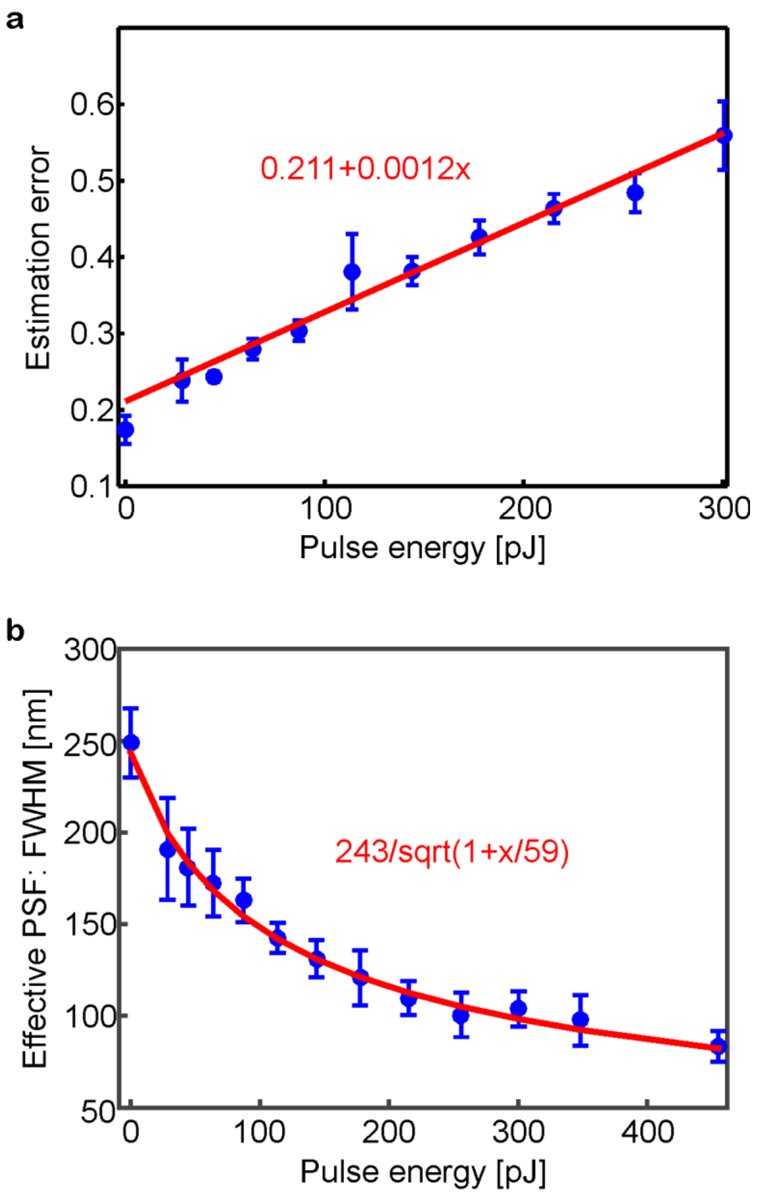


**Supplementary Figure 4: Dependence of uncertainty of molecule mapping on depletion power in STED microscopy**

Immobilized dsDNA (labelled with ATTO 647N) is measured under different STED illumination. (**a**) The error (relative standard deviation/RSTD) of the estimation (based on the theory in supplementary note) is plotted against the STED laser pulse energy. Experimental conditions are kept except for different STED illumination pulse energy. The red line is the linear fit to the data, with parameters and model indicated by red text. (**b**) The measured FWHM of the effective PSF is plotted with different STED illumination pulse energy. The PSF is measured on the average of over 50 scans on single ATTO 647N labeled dsDNA immobilized on surface. The red line is the fitting curve to the measured effective PSF to the square root law of resolution scaling in STED microscopy[1]. Blue dots and error bars are mean and standard deviation (n=9).

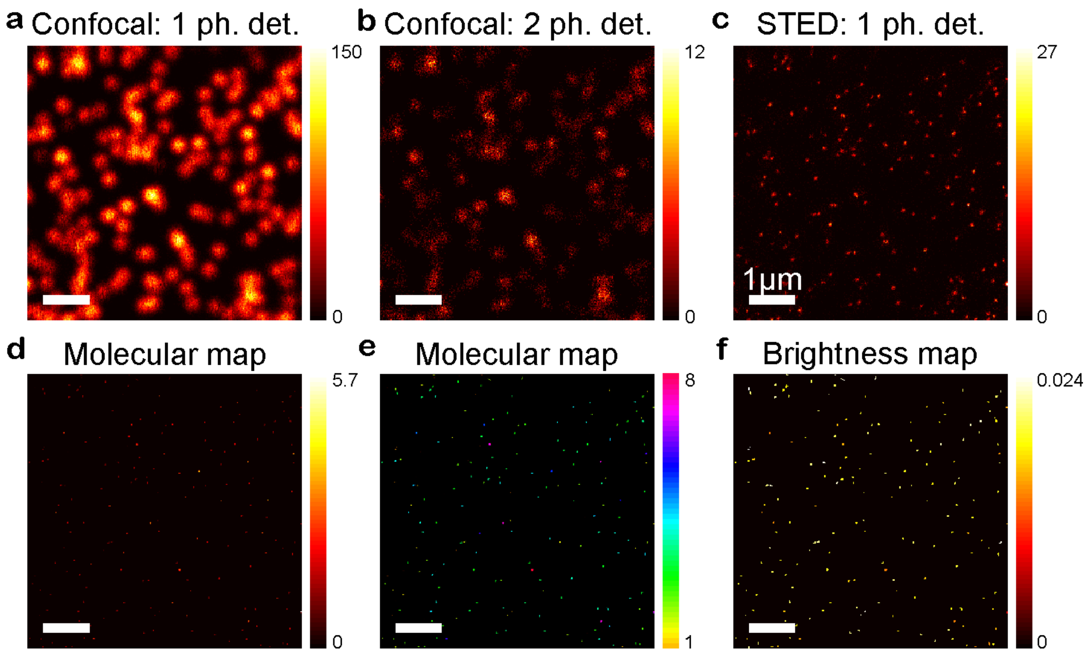


**Supplementary Figure 5: Mapping molecule distributions of immobilized dsDNA in the combination of confocal and STED microscopy through analysis of coincident photon detection**

ATTO 647N labelled dsDNA is immobilized on glass surface and measured with confocal and STED microscopy. Molecule mapping is achieved by analyzing the photon statistics of confocal and STED recording. (**a**,**b**) 1- and 2-photon detection confocal images of dsDNA on surface. (**c**) 1-photon detection STED image of the same area as **a**. (**d**,**e**) Molecular map based on the photon statistics of **a**, **b** and **c**. The color map of **d** codes the number of molecules at each pixel; the color map of **e** codes the number of molecules within each cluster. The segmentation of the clusters is processed with the built in watershed function in MATLAB[2]. (**f**) Molecular brightness map based on the photon statistics of **a**, **b** and **c** (only indicated where there are molecules). Scale bars: 1 μm

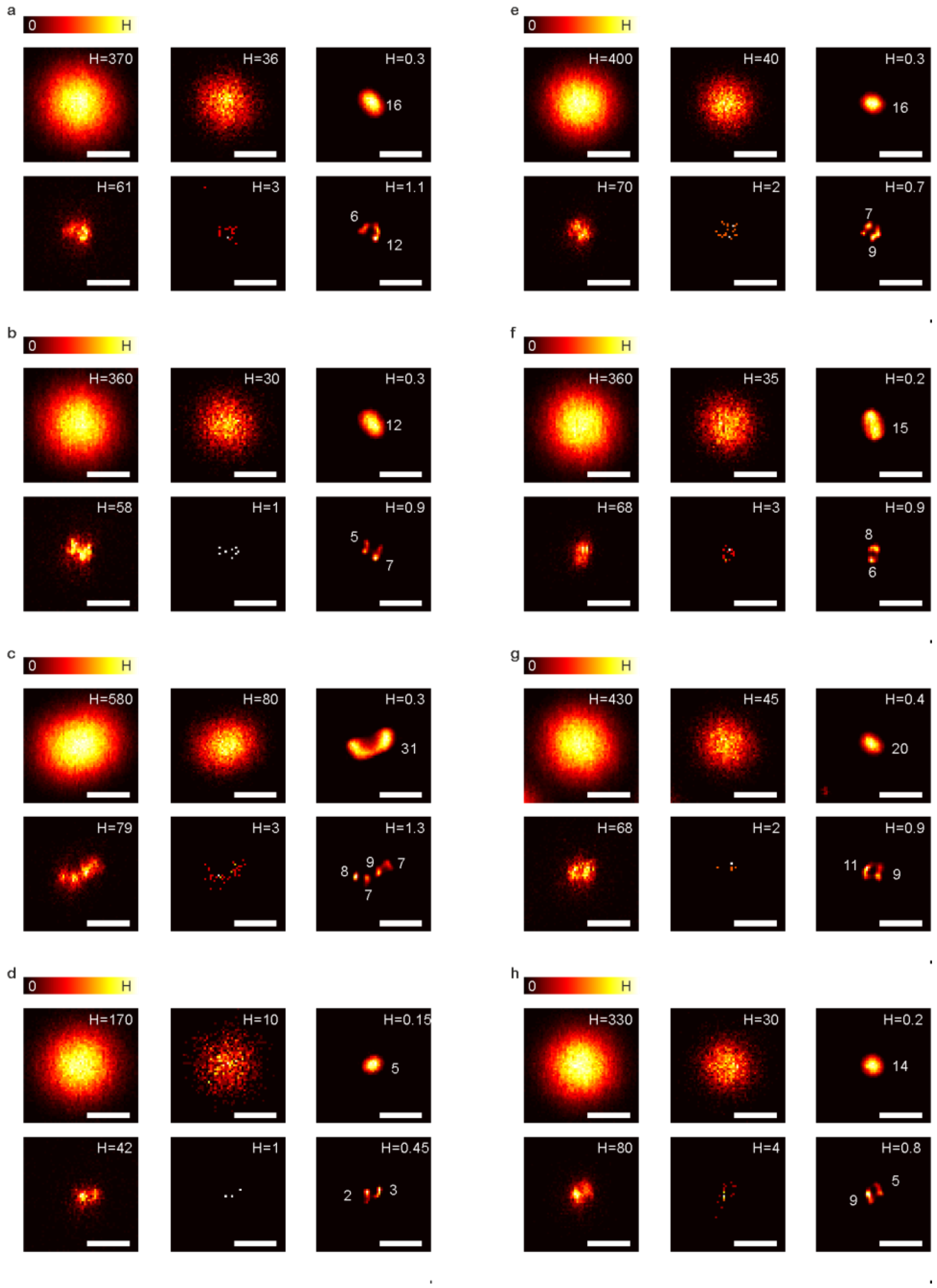

**Supplementary Figure 6: Mapping the number of molecules on DNA Origami**

DNA origami with up to 24 ATTO 647N was sparsely immobilized on the cover slip and measured by confocal and STED microscopy (see details in methods). (**a-h**) 8 examples of DNA origami. Top rows: Confocal 1- and 2- photon detection images and the corresponding estimated molecular map (from left to right). Bottom rows: STED 1- and 2-photon detection images and the estimated molecular map based on both confocal and STED measurements (from left to right). The number of molecules in each cluster is labeled at the vicinity. Scale bars: 200 nm.

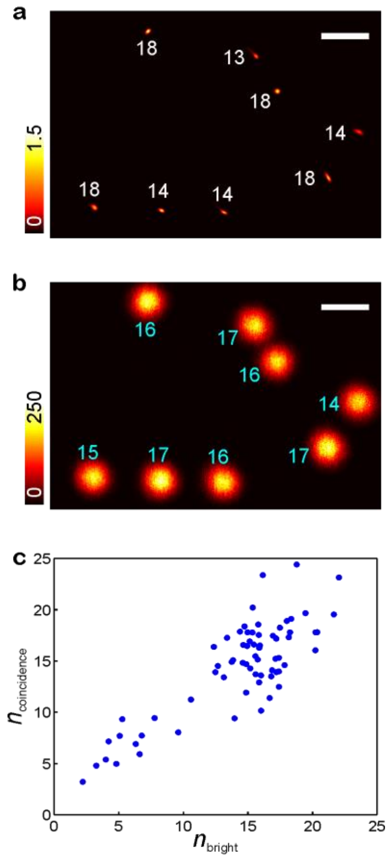


**Supplementary Figure 7: Comparison of molecular counting by photon statistics and molecular brightness on DNA origami with confocal microscopy**

DNA origami with up to 24 ATTO 647N was sparsely immobilized on the cover slip and measured by confocal microscopy. (**a**) The estimated molecular concentration $n$ based on photon statistics. The number of molecules in each DNA origami is the summation of the segmentation based on watershed algorithm[2] and labeled at the vicinity of the coordinate of the DNA origami. (**b**) The number of molecules in each DNA origami estimated by molecular brightness. Scale bars: 500 nm. (**c**) The scatter plot of the estimated number of molecules by coincidence photon detection $n_{coincidence}$ and that by dividing the intensity of the isolated DNA origami with molecular brightness $n_{bright}$. The method based on coincidence photon detection is described in the main text and supplementary note. The method based on molecular brightness is done as follows: the average molecular brightness is given by the former estimation method; the intensity of an isolated DNA origami is summed up and divided by the average molecular brightness and then divided by the summation of the PSF (normalized to the center). 69 isolated DNA origami are analyzed.

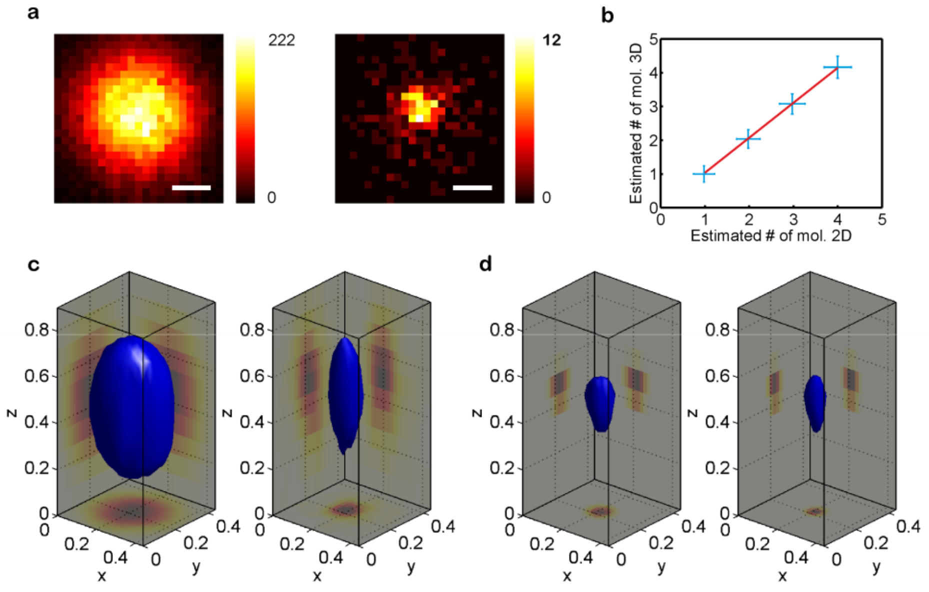


**Supplementary Figure 8: Comparison of 2D and 3D molecular counting of single dsDNAs**

dsDNA is immobilized on the glass surface and imaged on multiple z positions (100 nm distance between two immediate stacks) in both confocal and STED microscopy. (**a**) 2D image of a single dsDNA: confocal (left) and STED (right). The color code represents the event counts. Scale bars: 100 nm. (**b**) Estimated number of molecules in single dsDNAs from 2D and 3D imaging. The error bars represent the standard deviation and the red line is the linear fit to the mean value of the estimation (y=1.037x). (**c**) 3D imaging of the same dsDNA as that in **a**: fluorescence intensity isosurface (70% of the maximum intensity) with summation projections of fluorescence intensity on each dimension. Left: confocal; right: STED. (**d**) Estimation of the number of molecules of the same dsDNA of **a** and **c**: The isosurfaces, which embraces 70% of the molecules, are plotted with the summation projections on each dimension. Pixel size is 20 nm in lateral dimensions. Pixel dwell time is 300 μs (2D) and 100 μs (3D).

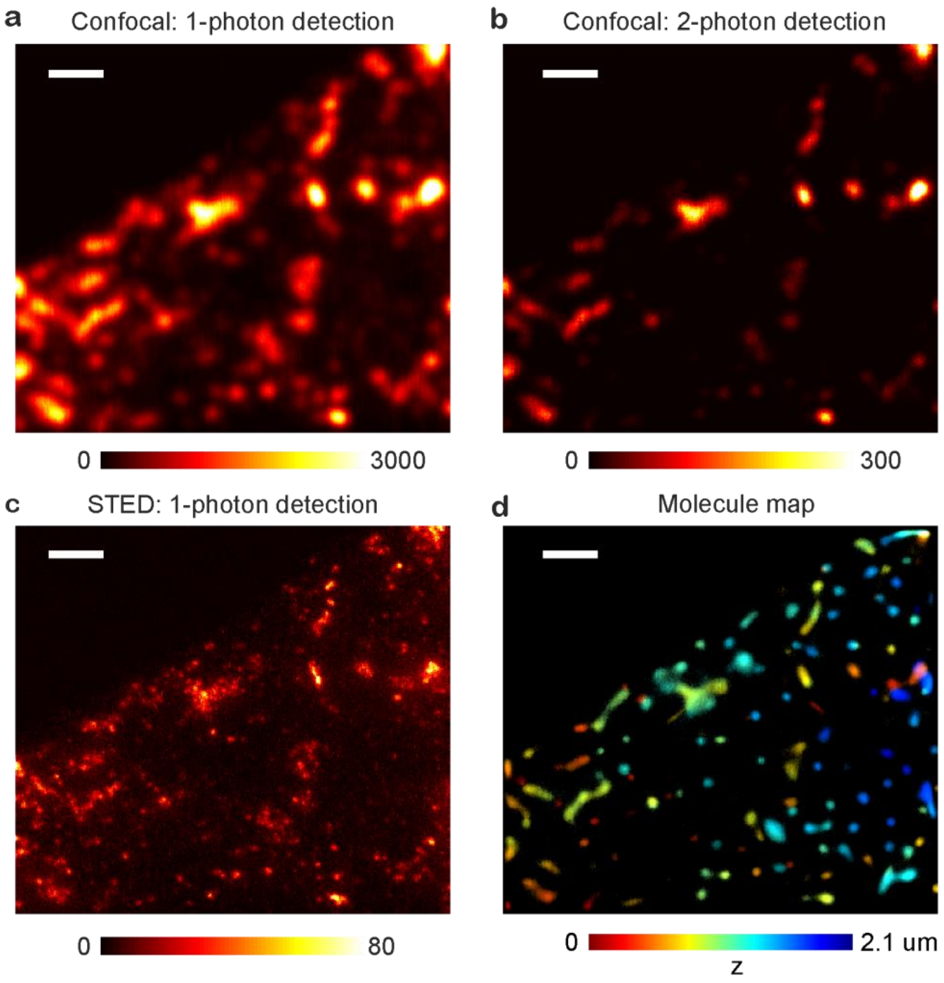


**Supplementary Figure 9: 1- and 2-photon detection events and the resulting molecule map of transferrin receptors in HEK293 cells**

Transferrin receptors stained with aptamer in HEK293 cells (refer to **Methods** in the main text for detailed sample preparation). (**a**, **b**) Axial summation of 1- and 2-photon detection events by confocal microscopy. The color code represents the detection event counts. (**c**) Axial summation (6 stacks cover 0.9 µm) of 1-photon detection events by STED. The color code represents the detection event counts. (**d**) Molecular map calculated obtained from photon statistics of **a** and **b**. The color represents the axial position (z) of the molecules. Scale bars: 1 µm.

a

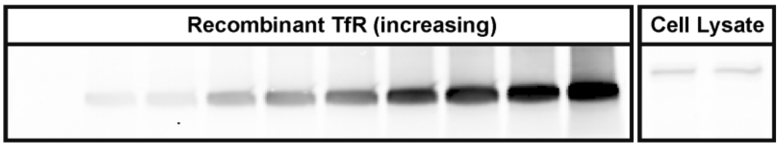


b

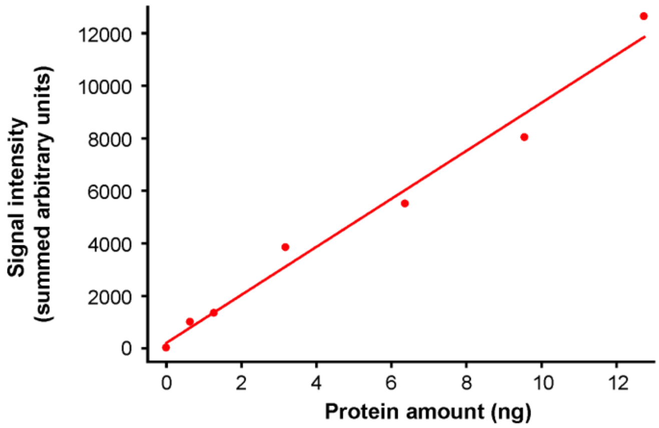


c

| Quantification per average HEK293 cell | |
|---|---|
| Total volume (femtoliters) | 3,625 ± 219.8 |
| Cytoplasmic volume (femtoliters) | 2,664 ± 201.3 |
| Total number of TfR | 175,130 ± 15,554 |
| Number of surface TfR (37 ± 2.2 % of total) | 64,798 ± 6,920 |
| Number of intracellular TfR (63 ± 3.8 % of total) | 110,332 ± 11,806 |

**Supplementary Figure 10: Quantitative western blot of transferrin receptors (TfR) in HEK293 cells**

(**a**) Western blot of the transferrin receptors from cell lysate compared to the recombinant transferrin receptor standard loaded in increasing amounts from 0 to 50 ng. Fetal calf serum was added to the recombinant protein in amounts matching the total protein concentration in the HEK293 cell lysate in order to have equal total protein concentration in all lanes. (**b**) A standard curve was created based on the increasing recombinant TfR signal in western blots. Linear regression was applied on the standard curve to determine the absolute amount of the TfR in the cell lysate. (**c**) Cytoplasmic volume and total volume of HEK293 cells were calculated experimentally on stained cells (see Methods), and the cellular distribution (internalized vs. cell surface) of TfR was determined by immunostaining and the resulting values are expressed as percentages (see Methods). These values were used to estimate the average number of TfR per unit of volume (femtoliter), as well as the number of receptors present on the cell surface and inside the cell. The values are mean ± SEM (n=3).

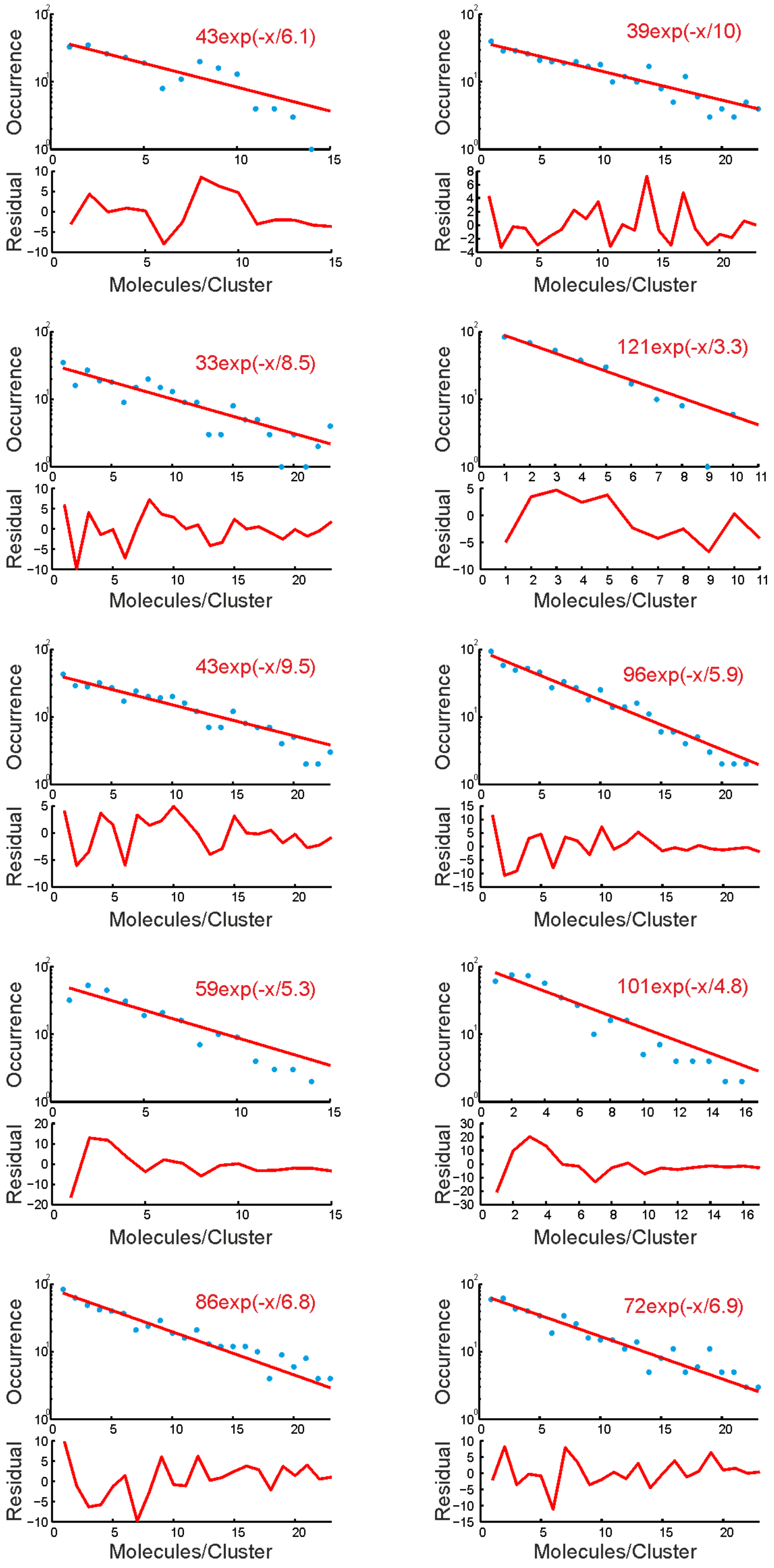


**Supplementary Figure 11: Histograms of the number of transferrin receptors (TfR) in single clusters**

Histograms of the number of TfR in single clusters from 10 different scanning regions of different cells. Red line: exponential distribution fitting to the occurrences (blue dots) of the molecules in each cluster with the fitting model and resulting parameters indicated in red text. The lower parts in each panel are the residuals of the fitting from the corresponding histograms. Analysis of clustering is performed with built in watershed algorithm provided in MATLAB[2]. The TfR clusters with high number (>24) of molecules are not considered due to the potential risk of overlapping clusters under the given resolution of the STED setup. The fitting range is chosen from 1 to the occurrences before the first zero instances in each panel.

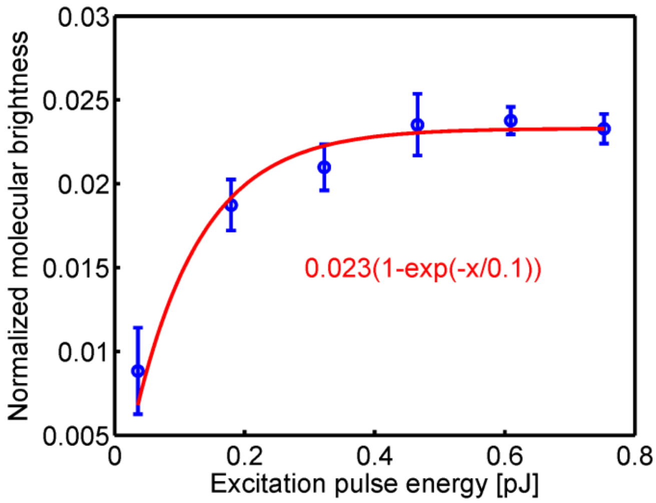


**Supplementary Figure 12: Excitation saturation of the molecular brightness**

Immobilized dsDNA labeled with up to 4 ATTO 647N molecules was imaged in a confocal microscope. The molecular brightness is estimated by recording the photon statistics. The saturation behavior of molecular brightness is plotted against increasing excitation pulse energy: Blue circles are mean values with standard deviation above and below. Each point is based on 3 regions of more than 200 molecules. The red line is the fit of an exponential saturation to the data. The fitting model and the resulting parameters are indicated by the red text.

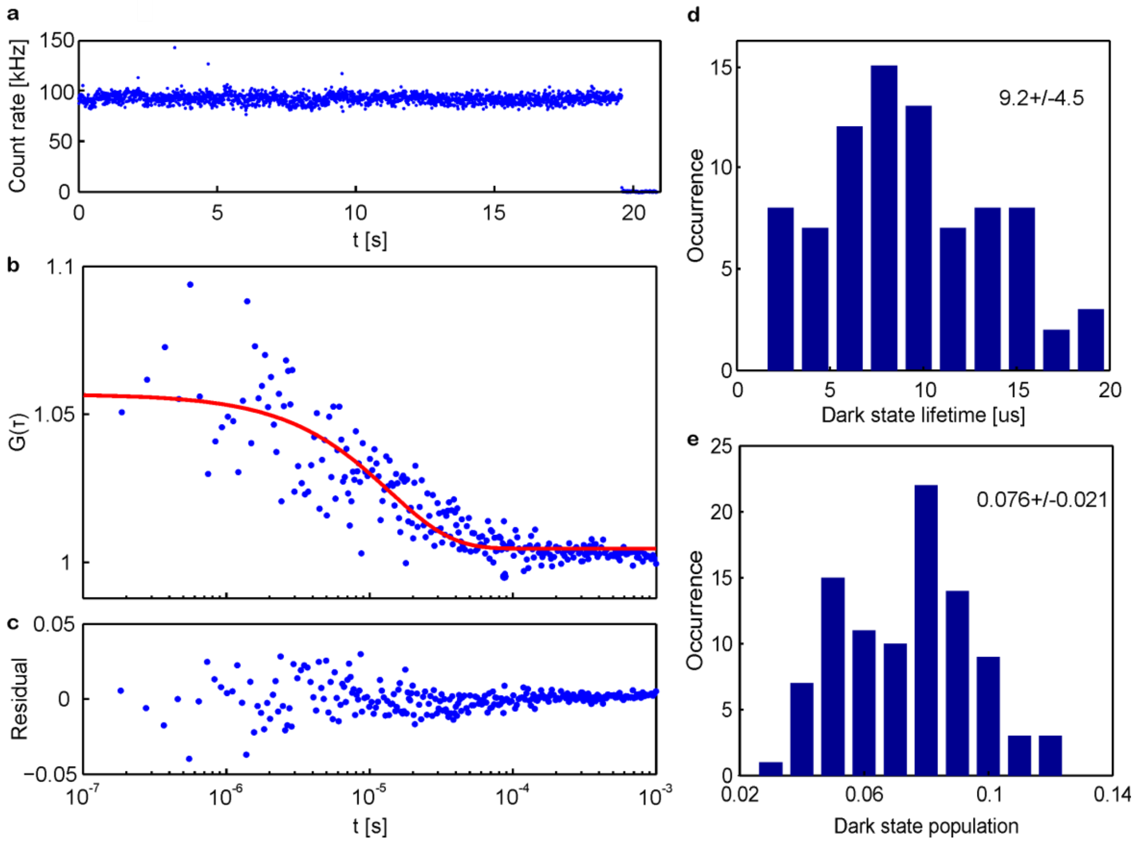


**Supplementary Figure 13: The population and lifetime of the dark state of single ATTO 647N molecules**

Immobilized dsDNA labeled with ATTO 647N molecules was imaged in a confocal setup. The dsDNAs with a single label were identified by observing a single bleaching step. The population and lifetime of the dark state, *e.g.* triplet state, of single ATTO 647N molecules was examined. (**a**) A typical time resolved fluorescence intensity trace of dsDNA labeled with a single ATTO 647N molecule. (**b**) Cross-correlation (blue dots) is calculated from the fluorescence intensity trace (0 - 18 s) in **a**. Red line: fit of the correlation curve with one population. (**c**) The residual of the fitting in **b**. (**d**,**e**) The histograms of the lifetime (**d**) and population (**e**) of the dark state from 83 molecules. The dark state lifetime is 9.2+/-4.5 µs (mean+/-standard deviation) and the dark state population is 0.076+/-0.021 (mean+/-standard deviation). The fitting model in **b** is $g(\tau) = 1 + T_{eq}/(1 - T_{eq})\exp(-\tau/\tau_T)$, with $T_{eq}$ the dark population at equilibrium and $\tau_T$ the dark state lifetime[3].

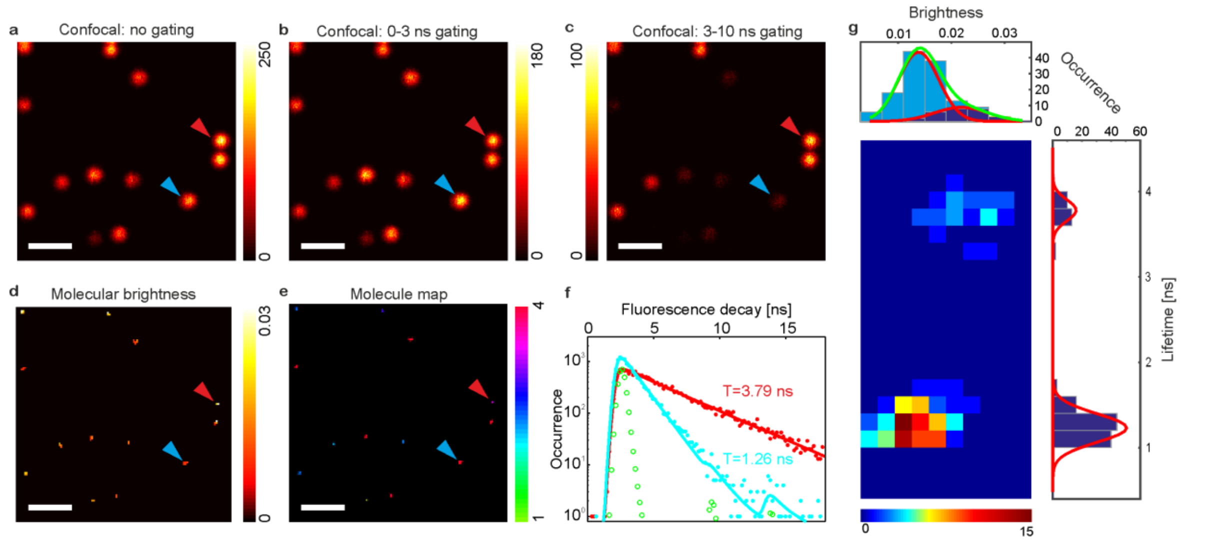


**Supplementary Figure 14: Differentiating single dsDNA with fluorescence lifetime & molecular brightness**

Mixture of dsDNA labelled with ATTO 647N or Cy5 are immobilized on the surface. Molecular brightness of each dsDNA is calculated by photon statistics. Fluorescence lifetime of isolated dsDNA spots is also found because our confocal recording is time correlated single photon counting measurements as well. ATTO 647N and Cy5 labelled dsDNA differ in molecular brightness and fluorescence lifetime due to the differences in their properties, such as quantum yield and extinction coefficient.

(**a**,**b**,**c**) An example of confocal image of the immobilized dsDNA (**a**: without time gating, **b**: 0~2 ns time gating, **c**: 2~10 ns time gating). (**d**,**e**) Molecular brightness **d** and molecule map **e** obtained from the photon statistics of confocal recoding in **a**. Color coding in **e** represents the number of molecules in the dsDNA cluster. (**f**) Fluorescence decay (cyan & red) of two single dsDNAs. Green circles represent the instrument response scaled to the same height as the red curve. Solid lines are single exponential decay fit to the fluorescence decays[4] with the lifetime information indicated by text in the same color. The locations of the corresponding dsDNA are indicated in **a**-**e** with triangles in the same colors. (**g**) Histograms of fluorescence lifetime and molecular brightness of the isolated dsDNA spots. Top: Histogram of the molecular brightness with two Gaussian peak fits; the parameters are (0.014, 0.005) and (0.022, 0.007) respectively. The green line is the summation of the two fits (red lines). Bottom left: 2D histogram of the molecular brightness and fluorescence lifetime. Color codes the number of occurrences. Bottom right: Histogram of the fluorescence lifetime with two Gaussian peak fits (red lines); the corresponding parameters are (1.23, 0.23) and (3.78, 0.17) respectively. Scale bars: 1 µm.

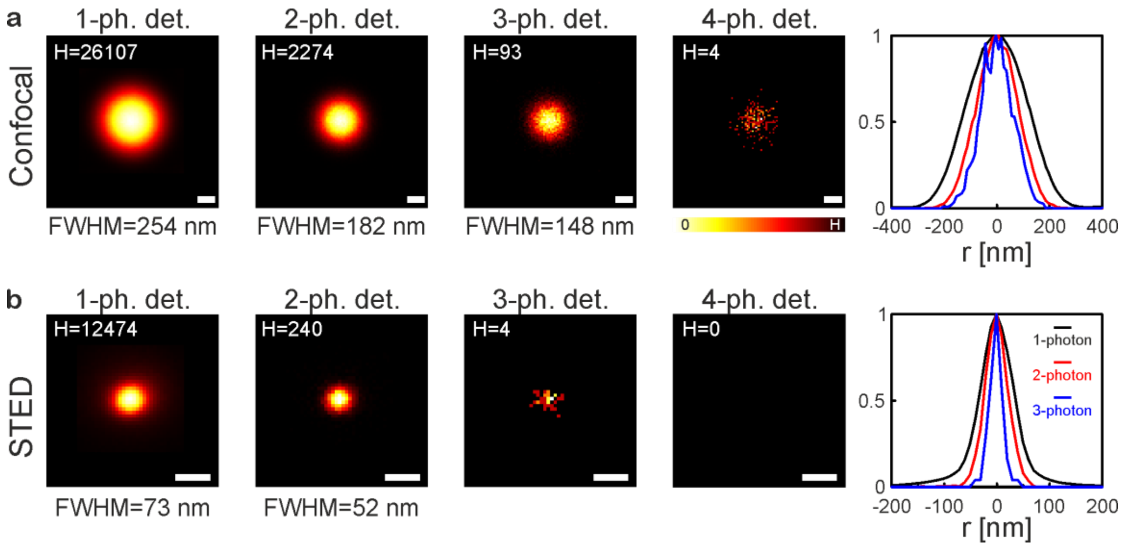


**Supplementary Figure 15: The point spread functions of 1-, 2-, 3- and 4-photon detection events in the confocal and STED microscopes measured on DNA origami**

DNA origami with up to 24 ATTO 647N in two lines of 41 nm spacing was sparsely immobilized on the cover slip and measured by (**a**) confocal (overlay of 64 single DNA origami images) and (**b**) STED microscopy (overlay of 114 single DNA origami images). The normalized line profiles crossing the center of the PSFs are on the right most; black: 1-photon, red: 2-photon and blue: 3-photon. The bright DNA origami probe the point spread functions of 1-, 2-, 3- and 4-photon detection events[5]. For all overlays allowing a good fit with a 2D Gaussian peak, the resulting full widths at half maximum ($\Delta r$) are displayed. H is the maximum value (counts) of the pseudocolor intensity scale. Scale bars: 100 nm.

# Supplementary Note 1

**Supplementary Note 1: Identifying the distributions of the number of molecules $\boldsymbol{n}(\boldsymbol{r})$ and their brightness $\boldsymbol{p}(\boldsymbol{r})$ from fluorescence photon emission statistics.**

Here we derive the theory underlying the photon statistics analysis and describe the procedure of extracting the number of molecules and the distribution of molecular brightness. Then, we discuss the uncertainty of the estimation on the number of molecules and the molecular brightness of a single cluster. In the end, we extend the derived model and reconstructing procedure to the case of combining two imaging modes, which is confocal and STED in our implementation.

**The probability to observe two-photon detection from the same molecule by single illumination pulse**

Here we calculate the probability to observe 2-photon detection from the same molecule by one illumination pulse.

We suppose that $I(t)$ is the laser intensity profile over time $t$, the photon emitter does not change its properties over time and the excited state population follows a single exponential decay as $\exp(-kt)$, $k$ is the decay rate of the excited state of the photon emitter including both the radiative and non-radiative decay rate, the probability of observing two decays is

$$\begin{aligned} P^{(2)} &= \sigma^2 k^2 \int_0^\infty I(t) \int_t^\infty \exp\big(-k(s-t)\big) \int_s^\infty I(u) \int_u^\infty \exp\big(-k(v-u)\big)\, dv\, du\, ds\, dt \\ &= \sigma^2 k \int_0^\infty \int_t^\infty \int_s^\infty I(t) I(u) \exp(-k(s-t))\, du\, ds\, dt, \end{aligned} \tag{1}$$

where $\sigma$ is the absorption cross section of the photon emitter.

For simplicity, we take the laser intensity $I(t)$ as a rectangular function with a width of $a$ and max intensity $l$ as given by

$$I(t) = \begin{cases} l, \text{if } 0 \leq t < a \\ 0, \text{if } t \geq a. \end{cases} \quad (2)$$

If we insert Eqn. (2) into Eqn. (1), by taking into account that $a \ll \tau_s$, Eqn. (1) turns into

$$P^{(2)} = \frac{1}{6}(\sigma l a)^2 a k, \quad (3)$$

where $la$ is the energy of the illumination pulse.

Therefore, the probability to detect two photons from one photon emitter by one illumination pulse is $\widehat{P^{(2)}} = \alpha^2\gamma^2 P^{(2)}$, $\alpha$ is the collection efficiency of the instrument and $\gamma$ is the quantum yield of the photon emitter.

If we compare $\widehat{P^{(2)}}$ to the square of typical one photon detection probability $\widehat{P^{(1)}} = \alpha\sigma\gamma l a$,

$$\frac{\widehat{P^{(2)}}}{\widehat{P^{(1)}}^2} = \frac{ak}{6} = \frac{a}{6\tau_s}. \quad (4)$$

Here, $\tau_s$ is the lifetime of the excited state of the photon emitter and $\tau_s = 1/k$.

That is, the probability to observe two photons from the same molecule is still negligible since $a \ll \tau_s$, even compared to the case of detecting two photons from two different molecules with the same molecular properties. Similarly, the probabilities for detection of multiple photons from the same molecule by one illumination pulse are negligible as well. Therefore, we can assume that no more than one photon can be emitted and, then, detected from one photon emitter by one illumination pulse.

**Spatial coincidence counting model**

Our experimental conditions make sure that the duration of the illumination pulse is much shorter than the lifetime of the photon emitter. As proved before, the probability for a single molecule to emit more than one photon per illumination pulse is negligible. We suppose that $p_i$ is the molecular brightness of the $i^{th}$ molecule located at position $s_i, i = 1, \ldots, N$, $N$ is the overall number of molecules. It is governed by the excitation intensity and the detection

efficiency of the optical system, but also by the properties of the molecule, such as absorption cross-section, fluorescence quantum yield, and dipole moment orientation. If $h(r)$ is the point-spread function (PSF, invariant in space and normalized to 1 at the center) of the imaging system with $r$ as the coordination, the term $p_i h(r - s_i)$ is an expression for the fluorescence intensity of the $i^{th}$ molecule, *i.e.* the probability of molecule $i$ to contribute with one photon to the detection by one illumination pulse if the current scanning position is $r$. Therefore, the probabilities of the arrival of a single and of two photons at the detectors, denoted with $e_1(r)$ and $e_2(r)$, respectively, originating from any of the molecules, can then be expressed as:

$$
\begin{aligned}
e_1(r) &= \sum_i p_i h(r - s_i) \prod_{j \neq i} \left(1 - p_j h(r - s_j)\right) \\
e_2(r) &= \sum_i p_i h(r - s_i) \sum_{j > i} p_j h(r - s_j) \prod_{k \neq i,j} \left(1 - p_k h(r - s_k)\right).
\end{aligned} \tag{5}
$$

Here we exploited the fact that the fluorescence arrival of the molecules do not depend on each other. In the case of arrival of two photons, we explicitly require them to originate from two distinct molecules. Furthermore, the product terms in Eqns.(5) can be neglected when $\sum_i p_i h(r - s_i) \ll 1$ holds everywhere. We then obtain:

$$
\begin{aligned}
e_1(r) &\approx \sum_i p_i h(r - s_i) \\
e_2(r) &\approx \frac{1}{2}\left[\left(\sum_i p_i h(r - s_i)\right)^2 - \sum_i \left(p_i h(r - s_i)\right)^2\right].
\end{aligned} \tag{6}
$$

**Transition to molecular densities on a continuous grid**

To simplify the situation we denote $n(r)$ as the density of fluorophores and $p(r)$ as the molecular brightness of the molecules at position $r$ and assume that all molecules at the same position share the same molecular brightness. Although the molecular brightness can be influenced by the properties of the molecule, such as absorption cross-section, fluorescence quantum yield and dipole moment orientation, the variation of the brightness is ~ 15%[6] in aqueous solution. For example, the effect of the dipole moment orientation can be largely washed out due to the free rotation of the dye molecules in the space of a cone in solution[7]. Therefore, the assumption is not far from the reality. Additionally, the variation in molecular brightness beyond the resolution of the microscope is taken into account in this model. The sums in Eqns. (6) can then be expressed as convolutions:

$$\begin{aligned} &\sum_i p_i h(r - s_i) \rightarrow (pn) * h \\ &\sum_i \left(p_i h(r - s_i)\right)^2 \rightarrow (p^2 n) * h^2, \end{aligned} \tag{7}$$

whereby the star "$*$" denotes convolution and the arrow means replacement by the simplified convolution terms.

**Influence of detection geometry**

In reality, detectors may not be able to identify the number of the arriving photons after one illumination pulse because of the dead time of the detectors. Therefore, multiple detection channels are required to detect the simultaneously arriving photons. However, still multiple photons can reach the same detector after one illumination pulse and the information of the photon numbers are partially lost. This loss can be taken into account by a geometrical factor $\beta \leq 1$. Hence the probabilities $f_1(r)$ and $f_2(r)$ for obtaining 1- and 2-photon detection events, respectively are given by

$$\begin{aligned} &f_1(r) \approx e_1(r) \\ &f_2(r) \approx \beta e_2(r). \end{aligned} \tag{8}$$

When $m$ independent identical detection channels are used and each channel is able to detect only up to one photon, the factor $\beta = (m-1)/m$. Inserting Eqns. (6) into Eqns. (8) yields Eqns. (1) in the main text:

$$\begin{aligned} f_1(r) &= ((pn) * h)(r) \\ f_2(r) &= \frac{\beta}{2}\left(\left((pn) * h\right)^2 - (p^2 n) * h^2\right)(r). \end{aligned} \tag{9}$$

Note that the main approximation in Eqns. (9) is that the product of local molecular number density and the local molecular brightness is small, i.e. $(pn * h)(r) \ll 1$ at any $r$. If this condition is not fulfilled, contributions from higher orders of photon coincidences must be included. Under our experimental conditions, the number of background photons is low enough to be negligible in most cases. When the background has to be considered and is Poissonian distributed, Eqns. (**9**) and Eqns. (1) in the main text can be extended accordingly as

$$\begin{aligned} f_1(r) &\approx e^{-\xi} g_1(r) + e^{-\xi}\xi\left(1 - \frac{m-1}{m} g_1(r)\right) \\ &\approx \left(1 - \frac{2m-1}{m}\xi\right) g_1(r) + \xi \\ f_2(r) &\approx \frac{m-1}{m} e^{-\xi} g_2(r) + e^{-\xi}\xi\left(\frac{m-1}{m} g_1(r) - \frac{m-1}{m}\frac{2m-1}{m} g_2(r) + \frac{2(m-1)}{m^2} g_2(r)\right) \\ &\approx \left(1 - \frac{3m-3}{m}\xi\right)\frac{m-1}{m} g_2(r) + \frac{m-1}{m}\xi g_1(r). \end{aligned} \tag{10}$$

Here, $g_1(r) = \left((pn) * h\right)(r)$, $g_2(r) = \frac{1}{2}\left(\left((pn) * h\right)^2 - (p^2 n) * h^2\right)(r)$ and $\xi$ is the mean value of the Poissonian background. We neglect the terms that are much smaller than $\xi$ and $p\xi$ in $f_1(r)$ and $p\xi$ and $p^2\xi$ in $f_2(r)$ in Eqns. (10) since $\xi \ll p(r) \ll 1$ under our experimental conditions. The background mainly includes the dark count of the detectors and scattered light from the sample in our experiments, which is typically ~ 1e-4/pulse or less. In the case of the 2D examples (dsDNA and DNA origami, Fig. 1 & 2 in the main text), the background is so low that it can be neglected without influencing the estimation of the number of molecules.

**Solving for $\boldsymbol{n}(\boldsymbol{r})$ and $\boldsymbol{p}(\boldsymbol{r})$ using penalized least squares**

Suppose that $Y_1(r_j)$ and $Y_2(r_j)$ are the experimental observations of the 1- and 2-photon detection events, respectively, normalized by the number of illumination pulses $M$. That means $Y_1(r)$ and $Y_2(r)$ are the frequency of 1- and 2-photon detection events at each grid position $r_j$, respectively

$$Y_i(r_j) = f_i(r_j) + \Delta_i(r_j), \quad i = 1,2. \tag{11}$$

Here, $\mathrm{e}_{1,2}(\mathrm{r})$ are the uncertainties introduced by measurement errors. Since the 1- and 2-photon detection events are counting events with low probabilities and with $M$ times, $\Delta_{1/2}(\mathrm{r})$ will be dominated by shot noise. We also assume that the errors are approximately normally distributed, especially when M is large as in our experiments, which enables us to apply a constraint maximum likelihood framework which essentially means solving a least squares problem. Hence, in order to recover $n(r)$ and $p(r)$, we employ a penalized maximum likelihood estimator as the solution of the following constraint optimization problem:

$$\begin{aligned} &\text{minimize:} && \alpha_1\|f_1(n,p) - Y_1\|^2 + \alpha_2\|f_2(n,p) - Y_2\|^2 + \gamma\phi\,(p) \\ &\text{subject to} && n \geq 0 \text{ and } p \geq 0. \end{aligned} \tag{12}$$

Here, $\alpha_1$, $\alpha_2$ and $\gamma$ are positive weighting parameters and $\phi$ is a typical penalizing term[8]. We applied the Laplacian on the brightness ($\phi = \nabla^2 p$) in order to enforce smoothness in the brightness distribution. With the value of $\gamma$ appropriately chosen, this penalization sufficiently stabilizes the solution of Eq. (12), preventing strong spatial oscillations in brightness on scales smaller than the full width at half maximum of the effective PSF. The weighting parameters $\alpha_{1,2}$ are chosen such that both least square residuals are on a similar scale. We found that $a_i = 1/\sum_j Y_i(r_j)$ largely achieves this goal. In order to incorporate the non-negativity constraints we substituted $n(r_j)$ and $p(r_j)$ by squared variables $m^2(r_j)$ and $q^2(r_j)$, respectively, and solved Eqn. (12) for $m(r_j)$ and $q(r_j)$ instead.

For the least-squares minimization of Eqn. (12), the gradient algorithm and other established methods are available. We chose the image reconstruction with the fast proximal gradient (FISTA) algorithm[9] of Beck and Teboulle which is numerically more efficient than the classical gradient algorithm. As starting values for $n(r_j)$ we choose a deconvolved 1-photon

detection image scaled by the average molecular brightness which was obtained assuming a constant molecular brightness over the whole image region (see below). Here, we chose 10 iterations of the Richardson-Lucy deconvolution[10] through our evaluation of experimental data. Usually 100-300 iterations were required to achieve stable solution and sufficient minimization. An automatic way to stop the iterations is suggested by Bissantz N, Mair BA and Munk A[11]. The implicit step corresponds to smoothing with the inverse of the Laplacian operator and therefore guarantees that $p(r)$ remains smooth during the iterative process and increases stability of the numerical minimization scheme. Instead of penalization with the Laplacian operator in FISTA, an explicit smoothing step of $p(r)$ (by convolution with a Gaussian smoothing kernel) in each iteration is also able to reject the strong variation of it. It should be possible to use other types of methods, such as Newton type methods[12] or iteratively reweighted least squares algorithm[13], to find the solution of Eqns. (12) as well.

**Direct solution for areas of constant molecular brightness**

In the case of an isolated (i.e. without molecules close to the border) area $A$ of constant molecular brightness $p(r_j) = p_0$, for $r_j \in A$, we can solve for the molecular brightness as well as for the total number of molecules $n_0 = \sum_{r_j \in A} n(r_j)$ within this area explicitly. In this case, summing the convolution terms in Eqns. (9) gives simple products:

$$\begin{aligned} \sum_{r_j \in A} pn * h &= p_0 n_0 H_1 \\ \sum_{r_j \in A} (p^2 n) * h^2 &= p_0^2 n_0 H_2, \end{aligned} \tag{13}$$

where the sums over the PSF are abbreviated by $H_i = \sum_{r_j \in A} h^i(r_j)$, $i = 1,2$. Eqns. (9) then uniquely determine $p_0 n_0$ as well as $p_0^2 n_0$ and $n_0$ and $p_0$ can be solved:

$$n_{est} = \frac{H_2}{H_1^2} \frac{\left(\sum_{r_j \in A} Y_1\right)^2}{\sum_{r_j \in A} Y_1^2 - \frac{2}{\beta} \sum_{r_j \in A} Y_2}$$
$$p_{est} = \frac{H_1}{H_2} \frac{\sum_{r_j \in A} Y_1^2 - \frac{2}{\beta} \sum_{\in \subset A} Y_2}{\sum_{r_j \in A} Y_1}. \tag{14}$$

Here, $n_{est}$ and $p_{est}$ are the estimation of and $n_0$ and $p_0$. If the PSF $h$ can be approximated by a Gaussian peak function, the terms $H_i$ in Eqns. (14) can be simplified to simple factors. Provided the variation of molecular brightness within an isolated region of interest is small (~20%), the expressions in Eqns. (14) can be used to compute an initial estimate of the total number and the average brightness of the molecules within this region.

**Uncertainty of estimating the number and brightness of a single, isolated cluster of molecules**

We use Eqns. (14) to derive a measure for the uncertainty of the estimated number $n_{\mathrm{est}}$ and brightness $p_{est}$ of $n_0$ molecules in a single, isolated cluster with brightness $p_0$. Then, Eqns. (**9**) can be simplified to

$$f_1(r) = n_0 p_0 h(r)$$
$$f_2(r) = \frac{\beta}{2} n_0 (n_0 - 1) p_0^2 h^2(r). \tag{15}$$

The observed number of 1- and 2-photon detection events are the experimental realizations of multinomial distributed random variables with $M$ repeats, with probabilities $f_{1/2}(r)$. Because these probabilities are typically very small ($f_{1/2}(r) \ll 1$), we approximate the variances of the observed 1- and 2-photon detection events by $Mf_{1,2}(r)$. Therefore, the normalized number of 1- and 2-photon detection events $Y_{1,2}(r)$ have variances

$$\mathbb{V}\mathrm{ar}\left(Y_1(r)\right) \cong \frac{f_1(r)}{M} = \frac{n_0 p_0 h(r)}{M} \tag{16}$$

$$\mathbb{Var}(Y_2(r)) \cong \frac{f_2(r)}{M} = \frac{\beta n_0(n_0-1)p_0^2 h^2(r)}{2M}.$$

Without any long lasting historical effects (e.g. molecular blinking and bleaching) the observations at different positions $r$ are independent. Furthermore, the correlation between $Y_1(r)$ and $Y_2(r)$ is negligible, because $f_{1/2}(r) \ll 1$. Therefore, the error propagation of $n_{est}$ and $p_{est}$ in Eqns. (14) can be expressed by $\mathbb{Var}(Y_1(r))$ and $\mathbb{Var}(Y_2(r))$ in Eqns. (16). As $np \ll 1$, all terms containing $\mathbb{Var}(Y_1(r))$ are dominated by those containing $\mathbb{Var}(Y_2(r))$ and therefore can be neglected. Consequently, the simplified variant results in

$$\begin{aligned} \mathbb{Var}(n_{\mathrm{est}}) &\cong \frac{2}{\beta}\frac{n_0(n_0-1)}{p_0^2 M H_2} \\ V\mathbb{ar}(p_{\mathrm{est}}) &\cong \frac{2}{\beta}\frac{(n_0-1)}{n_0 M H_2}. \end{aligned} \tag{17}$$

The relative standard deviations (RSTD) are the same for the estimation of the number of molecules $n_{est}$ as well as for that of the molecular brightness $p_{est}$:

$$\frac{\sqrt{\mathbb{Var}(n_{\mathrm{est}})}}{n_0} = \frac{\sqrt{\mathbb{Var}(p_{\mathrm{est}})}}{p_0} \cong \sqrt{\frac{2(n_0-1)}{\beta p_0^2 n_0 M H_2}}. \tag{18}$$

The fact that the RSTDs are inversely proportional to $p_0$ makes it a critical parameter. Therefore, it suggests choosing a bright fluorophore with high quantum yield. Also good collection efficiency of the instrument is preferred. The RSTDs are also inversely proportional to $\sqrt{M}$, which is closely related to the pixel dwell time, so that a stable fluorophore is also preferred. If a (future) detector is able to identify the number of detected photons directly or does not have any dead time, $\beta$ can reach its limit 1.

Interestingly, the RSTDs are small for small numbers $n_0$ and asymptotically independent on $n_0$ when $n_0$ is large. In the ideal case that fluorophores do not bleach, a smaller effective focal volume, *e.g.* in the case of STED microscopy, will embrace fewer fluorophores and decrease the relative estimation error on both $n_0$ and $p_0$. The effect is significant when $n_0$ is close to 1.

## Combined confocal and STED measurements

If confocal and STED measurements are combined, all equations are doubled to accommodate for both imaging modes. Eqns. **(9)** turn into

$$
\begin{aligned}
f_{1,c}(r) &= (pn) * h_c \\
f_{2,c}(r) &= \frac{\beta}{2}\left(\left((pn) * h_c\right)^2 - (p^2 n) * h_c^2\right) \\
f_{1,s}(r) &= \varepsilon(pn) * h_s \\
f_{2,s}(r) &= \frac{\beta}{2}\varepsilon^2\left(\left((pn) * h_s\right)^2 - (p^2 n) * h_s^2\right).
\end{aligned}
\tag{19}
$$

Here, $f_{1,c}(r)$ and $f_{2,c}(r)$ are the 1- and 2-photon detection probabilities of the confocal microscopy, respectively; $h_c$ is the effective PSF of confocal microscopy. $f_{1,s}(r)$, $f_{2,s}(r)$ and $h_s$ are the counterparts of STED microscopy. In comparison to Eqns. (2) in the main text, $\varepsilon$ is additionally introduced as the molecular brightness ratio between confocal and STED microscopy. Theoretically, $\varepsilon = 1$, when the illumination pulse energy is kept the same in both confocal and STED measurements. In practice, the intensity minima of the STED light is not zero and $\varepsilon$, which was set to 0.5-0.7, is used to adjust the brightness in STED microscopy.

Analogously, the estimation function in Eq. (12) turns into

$$
\begin{aligned}
\text{minimize:}\quad & \alpha_{1,c}\left\|f_{1,c}(n,p) - Y_{1,c}\right\|^2 + \alpha_{2,c}\left\|f_{2,c}(n,p) - Y_{2,c}\right\|^2 \\
& + \alpha_{1,s}\left\|f_{1,s}(n,p) - Y_{1,s}\right\|^2 + \alpha_{2,s}\left\|f_{2,s}(n,p) - Y_{2,s}\right\|^2 + \gamma\phi\,(n,p) \\
\text{subject to}\quad & n \geq 0 \text{ and } p \geq 0.
\end{aligned}
\tag{20}
$$

Here, $\alpha_{1,c}$, $\alpha_{2,c}$, $\alpha_{1,s}$, $\alpha_{2,s}$ and $\gamma$ are positive weighting parameters and $\phi$ is the same penalizing term as in Eqn. (12). We choose $\alpha_{i,c} = 1/\sum_j Y_{i,c}(r_j)$ and $\alpha_{i,s} = 1/\sum_j Y_{i,s}(r_j)$, $i = 1,2$ to bring all least-square residuals to a similar scale, with $Y_{i,c}(r_j)$ and $Y_{i,s}(r_j)$ as the experimental realization of the 1- and 2-photon detection events normalized by the number of illumination pulses $M$. Again the fast proximal gradient algorithm (FISTA) can be applied to recover $n(r_j)$ and $p(r_j)$. The starting conditions and number of iterations can be chosen as in the non-combined case.

Similar to the non-combined case, if the molecular brightness remains constant $p(r_j) = p_0$, for $r_j \in A$, over an isolated area $A$, the total number of molecules $n_0 = \sum_{r_j \in A} n(r_j)$ and the average molecular brightness $p_0$ within this area can be solved as in Eqn. (14), and the uncertainty in estimation of the combined confocal and STED measurements can be deduced as in Eqn.(18). However, in practice, the estimation on overall number of molecules and molecular brightness on a region of interest and the relative uncertainty of the estimation from confocal images is mainly used because the measured PSF of STED is not as precise as that of confocal microscope.

**Supplementary References**


1 Harke, B. *et al.* Resolution scaling in STED microscopy. *Opt Express* **16**, 4154-4162 (2008).

2 Meyer, F. Topographic Distance and Watershed Lines. *Signal Process* **38**, 113-125, (1994).

3 Widengren, J., Rigler, R. & Mets, u. Triplet-state monitoring by fluorescence correlation spectroscopy. *J Fluoresc* **4**, 255-258 (1994).

4 Enderlein, J. & Erdmann, R. Fast fitting of multi-exponential decay curves. *Opt Commun* **134**, 371-378, (1997).

5 Hell, S. W., Soukka, J. & Haenninen, P. E. Two- and multiphoton detection as an imaging mode and means of increasing the resolution in far-field light microscopy. *Bioimaging* **3**, 65-69 (1995).

6 Ta, H., Kiel, A., Wahl, M. & Herten, D.-P. Experimental approach to extend the range for counting fluorescent molecules based on photon-antibunching. *Phys Chem Chem Phys* **12**, 10295-10300, (2010).

7 Stallinga, S. Effect of rotational diffusion in an orientational potential well on the point spread function of electric dipole emitters. *J. Opt. Soc. Am. A* **32**, 213-223, doi:10.1364/JOSAA.32.000213 (2015).

8 Eggermont, P. P. & LaRiccia, V. N. *Maximum Penalized Likelihood Estimation: Volume II: Regression*. (Springer, 2009).

9 Beck, A. & Teboulle, M. A Fast Iterative Shrinkage-Thresholding Algorithm for Linear Inverse Problems. *Siam J Imaging Sci* **2**, 183-202, (2009).

10 Richardson, W. H. Bayesian-based iterative method of image restoration. *J Opt Soc Am* **62**, 55-59 (1972).

11 Bissantz, N., Mair, B. A. & Munk, A. A multi-scale stopping criterion for MLEM reconstructions in PET. in *IEEE Nuclear Science Symposium Conference Record, 2006.* 3376-3379.

12 Hohage, T. & Werner, F. Iteratively regularized Newton-type methods for general data misfit functionals and applications to Poisson data. *Numer. Math.* **123**, 745-779, (2012).

13 Bissantz, N., Dümbgen, L., Munk, A. & Stratmann, B. Convergence analysis of generalized iteratively reweighted least squares algorithms on convex function spaces. *SIAM J. Optim.* **19**, 1828-1845, (2009).
